# Mapping of individual sensory nerve axons from digits to spinal cord with the transparent embedding solvent system

**DOI:** 10.1038/s41422-023-00867-3

**Published:** 2024-01-03

**Authors:** Yating Yi, Youqi Li, Shiwen Zhang, Yi Men, Yuhong Wang, Dian Jing, Jiayi Ding, Qingjie Zhu, Zexi Chen, Xingjun Chen, Jun-Liszt Li, Yilong Wang, Jun Wang, Hanchuan Peng, Li Zhang, Wenjing Luo, Jian Q. Feng, Yongwen He, Woo-Ping Ge, Hu Zhao

**Affiliations:** 1 State Key Laboratory of Oral Diseases, National Center for Stomatology, National Clinical Research Center for Oral Diseases, West China Hospital of Stomatology, Sichuan University, Chengdu, Sichuan China; 2 Department of Orthodontics, West China Hospital of Stomatology, Sichuan University, Chengdu, Sichuan China; 3 Chinese Institute for Brain Research, Beijing, China; 4 Department of Neurology, Beijing Tiantan Hospital, Capital Medical University, Beijing, China; 5 School of Basic Medical Sciences, Capital Medical University, Beijing, China; 6 Department of Oral Implantology, West China Hospital of Stomatology, Sichuan University, Chengdu, Sichuan China; 7 Department of Head and Neck Oncology, West China Hospital of Stomatology, Sichuan University, Chengdu, Sichuan China; 8 West China School of Public Health and West China Fourth Hospital, Sichuan University, Chengdu, Sichuan China; 9 Department of Orthodontics, Shanghai Ninth People’s Hospital, Shanghai Jiao Tong University School of Medicine; College of Stomatology, Shanghai Jiao Tong University; National Center for Stomatology; National Clinical Research Center for Oral Diseases; Shanghai Key Laboratory of Stomatology; Shanghai Research Institute of Stomatology, Shanghai, China; 10 Academy for Advanced Interdisciplinary Studies, Peking University, Beijing, China; 11 SEU-ALLEN Joint Center, Institute for Brain and Intelligence, Southeast University, Nanjing, Jiangsu China; 12 341 Hunnewell St Needham, MA, USA; 13 Texas A&M University, College of Dentistry, Dallas, TX USA; 14 Qujing Medical College, Qujing, Yunnan China

**Keywords:** Biological techniques, Bioinformatics

## Abstract

Achieving uniform optical resolution for a large tissue sample is a major challenge for deep imaging. For conventional tissue clearing methods, loss of resolution and quality in deep regions is inevitable due to limited transparency. Here we describe the Transparent Embedding Solvent System (TESOS) method, which combines tissue clearing, transparent embedding, sectioning and block-face imaging. We used TESOS to acquire volumetric images of uniform resolution for an adult mouse whole-body sample. The TESOS method is highly versatile and can be combined with different microscopy systems to achieve uniformly high resolution. With a light sheet microscope, we imaged the whole body of an adult mouse, including skin, at a uniform 0.8 × 0.8 × 3.5 μm^3^ voxel resolution within 120 h. With a confocal microscope and a 40×/1.3 numerical aperture objective, we achieved a uniform sub-micron resolution in the whole sample to reveal a complete projection of individual nerve axons within the central or peripheral nervous system. Furthermore, TESOS allowed the first mesoscale connectome mapping of individual sensory neuron axons spanning 5 cm from adult mouse digits to the spinal cord at a uniform sub-micron resolution.

## Introduction

The development of tissue clearing techniques was a major breakthrough in microscopy imaging. By processing biological samples with various chemicals, tissue could be made to be transparent and then could be imaged with a microscope. Current clearing methods can be largely divided into three major categories: aqueous methods, solvent-based methods and hydrogel-based methods.^1–9^ Most tissue clearing methods are based on similar chemical principles and are comprised of multiple steps including fixation, decalcification (for hard tissue), decolorization, delipidation, dehydration (for solvent-based clearing methods only) and refractive index (RI) matching.^1^

Achieving uniform high resolution throughout the entire sample is the current challenge for tissue clearing-based deep imaging methods. Many tissue clearing studies have demonstrated imaging of mouse whole-body samples at single-cell resolution.^2,5,7^ A lot of these studies claimed that micron-level structures could be visualized at a certain depth of the tissue. However, none of them had the confidence to claim that every fine structure in the tissue could be visualized with equal quality and resolution. In addition, structures of sub-micron level like single neural axons can only be visualized in superficial regions of the samples.

This could be attributed to insufficient tissue transparency and imperfect RI matching. With the minor optical aberrations that accumulate along the optical path, signal quality and intensity are inevitably lost in deep sample regions.^8,10^ The optical resolution deterioration is more severe in cleared peripheral organs as a result of their diverse tissue components. Additionally, objectives with a high numerical aperture (NA), which determines the physical resolution of the objectives, usually have a short working distance, which limits the imaging depth within the tissue.^11,12^ For this reason, mesoscale neural connectome mapping of rodent brains, a primary goal of tissue clearing technique, has never been accomplished by conventional tissue clearing methods.^13–17^ Moreover, no approach is available for mapping the complete projection of individual peripheral nerve axons in a rodent model. It is imminent to develop a new strategy to acquire volumetric images of uniformly high resolution within a large tissue sample.

We have thus designed the Transparent Embedding Solvent System (TESOS) method which incorporates tissue clearing, transparent embedding, sectioning and block-face imaging. We have combined TESOS with different microscopy systems to achieve uniform resolution throughout the entire sample. In combination with light sheet microscopy, TESOS resulted in an adult mouse whole-body imaging dataset at a uniform micrometer voxel resolution, which generated 70 terabytes of imaging data within 120 h. By combining with confocal microscopy, we achieved sub-micron resolution for large tissue samples on a scale of several centimeters on a side. Finally, we displayed the first complete mesoscale connectome mapping of individual sensory nerve axons from the peripheral nervous system (PNS) to the central nervous system (CNS).

## Results

### Development of the TESOS

The TESOS method can be largely divided into three steps including pretreatment, clearing and transparent embedding. The collection and pre-treatment of various samples are similar to the methods we described in our previous PEGASOS approach.^4^ In brief, after 4% paraformaldehyde (PFA) fixation, 25% *N*,*N*,*N*′,*N*′-Tetrakis (2-Hydroxypropyl) ethylenediamine (Quadrol) solution was used as the decolorizing reagent to remove heme.^4,6^ Gradient tert-butanol (tB) solutions were used for delipidation. tB-Quadrol (tB-Q) reagent was used for dehydration, which is composed of 70% tB and 30% Quadrol.

**Fig. 1:**
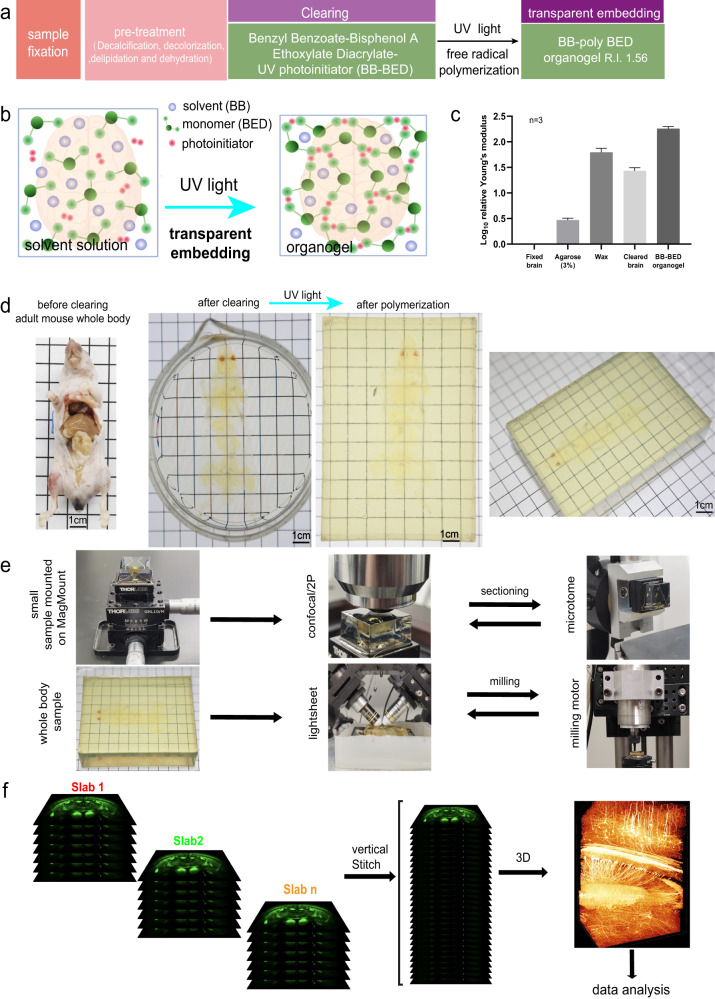
Technical pipeline of the TESOS method. **a** Sample processing steps of the TESOS method. **b** Formation of the organogel is the conceptual basis for transparent embedding. **c** Relative mechanical strength of various samples displayed based on log_10_ relative Young’s modulus values (*n* = 3). Young’s modulus value of a freshly fixed brain was normalized as 1. **d** Representative images of 6-week-old *Thy1-YFP-16* mice before clearing, after clearing and after polymerization. **e** Small samples were glued on a MagMount device and a rotary microtome was used for sectioning the surface. Large whole-body samples were directly glued within a chamber under a light sheet microscope for imaging in combination with a milling platform. **f** Data processing pipeline.

BB-BED clearing medium is a solvent-based clearing medium with high RI (1.552). BB-BED is composed of 47% (v/v) benzyl benzoate (BB), 48% (v/v) bisphenol-A ethoxylate diacrylate M_n_ 468 (BED) and 5% (v/v) Quadrol and then is supplemented with 2% (w/v) Irgacure 2959 as the UV initiator (Fig. 1a). BED is commonly used as a dental resin monomer due to its strength and low toxicity. It contains acrylate groups at both ends of the molecule. The BB-BED clearing medium rendered both soft- and hard-tissue organs highly transparent, including the brain, mandible, mouse paw with skin, vertebrae with bone and muscles, spleen, liver, heart, femur and even the whole body of mouse pups (Supplementary information, Fig. S1a, b).

Next, the BB-BED medium and the samples contained within it were irradiated with UV light for ~10 min. In the presence of the photoinitiator and UV light, BED molecules are cross-linked through free radical polymerization reactions to form transparent poly-BED polymer. BB solvent did not participate in the polymerization reaction and was dispersed among the polymer to form an organogel (RI, 1.56) (Fig. 1b). Importantly, after polymerization, samples remained transparent in the BB-poly BED organogel (Supplementary information, Fig. S1a, b). We defined this process as “transparent embedding”.

TESOS method could be implemented as either a passive immersion or active recirculation approach. Passive immersion approach was for small samples or organs.^4^ For hard-tissue organs, an additional decalcification treatment with EDTA solution was included before decolorization step. Typically, it took 8 days (soft tissue organs) or 12 days (hard tissue organs) to reach final transparency. An active recirculation approach was used for adult mouse whole-body samples (Fig. 1d).^4^ An additional melanin bleaching step was included in the pretreatment to decolorize skin pigment. This treatment could be applied to either agouti or black-skin mice, turning the skin pale or light-colored (Supplementary information, Fig. S1c, d). As tested in whole-body samples of *Thy1-YFP-16* mice, it reduced the YFP fluorescence within the skin by ~25% but had no significant impact on nerve signal intensity within the muscles (Supplementary information, Fig. S1e, f). Autofluorescence from both the skin and muscle reduced significantly after melanin bleaching treatment (Supplementary information, Fig. S1f). The entire background including the skin and all internal organs of C57BL/6J (BL6) mice could be turned into nearly invisible after clearing and maintained equally transparent after UV light polymerization (Fig. 1d).

The effect of clearing upon endogenous fluorescence was evaluated with *Thy1-EGFP-M* mouse brain slices and *Gli1-cre*^*ERT2*^*;Ai14* mouse intestine samples. At the end of either PEGASOS or TESOS clearing process, GFP and tdTomato fluorescence retained ~70% of the original intensity, with no significant difference between the two methods. The polymerization process has no significant impact on the GFP or tdTomato fluorescence (Supplementary information, Fig. S2a, b). Changes in transparency after transparent embedding were evaluated with *Thy1-EGFP-M* brain samples. Confocal images with comparable signal intensity and quality were acquired before and after polymerization (Supplementary information, Fig. S2c, d). Quantitative analysis reveals no significant change in signal intensity, indicating no substantial loss in transparency after the transparent embedding process (Supplementary information, Fig. S2e). Signal-to-noise ratios (SNRs) were calculated in images of samples either treated with PEGASOS or TESOS. The two methods showed similar SNRs at various imaging depths (Supplementary information, Fig. S2f).

The TESOS method also had a favorable effect on the mechanical strength of the samples. A transparently embedded brain was more than 150-fold stronger than a freshly fixed brain. In comparison, agarose embedding improved sample strength by ~3-fold and paraffin embedding improved brain strength by ~100-fold (Fig. 1c).

In brief, the TESOS method efficiently cleared tissue organs, and made them highly transparent without a loss in endogenous fluorescence and with improvements in tissue strength of more than 150-fold. Strong mechanical strength enables a tissue sample to be machined via either a sectioning or milling approach without causing distortion.

For small samples (<1 cm on a side), a sectioning strategy based on a rotary microtome and MagMount device was developed (Fig. 1e). The MagMount consisted of a goniometer and a magnetic kinematic base. Transparently embedded samples were glued onto the kinematic base top plate, which allowed high-precision repositioning with high repeatability as the sample was transferred between the microtome and microscope stage for imaging. The sample block surface was imaged like a regular flat slab with the image thickness being determined mainly by the objective working distance. Afterward, samples were transferred to the rotary microtome and sectioned at 5 µm/round to 10 µm/round to expose the next slab surface, which was then imaged. 10% of overlap was maintained between adjacent slabs for stitching reference. Imaging *Thy1-EGFP-M* brain samples showed that this strategy could significantly increase SNR in the deep regions (Supplementary information, Fig. S2f). It is highly flexible and can be easily combined with all upright confocal/two-photon (2P) microscopes without modification (Supplementary information, Fig. S2a).

Large samples consisting of multiple centimeters on a side were processed with the milling platform (Fig. 1e). The milling platform can stand alone or can be combined with an ASI Selective Plane Illumination Microscopy (SPIM) system to form a semi-automated whole-body imaging platform (Supplementary information, Fig. S2b). A high-speed diamond bur was used to remove the sample surface to the designated depth.

Tissue distortion after the sectioning or milling process was tested with various samples. Images were acquired at the same locations before and after sectioning/milling for transparently embedded brain, vertebrae and whole-head samples. Overlaying of pre- and post-processing images indicated that tissue organization and fine structures such as the neuronal dendrites remained unchanged (Supplementary information, Fig. S2c–h). The dimensional stability after the machining process enabled adjacent image slabs to be easily stitched together in the vertical dimension without the need for transformation algorithms (Fig. 1f).

### Whole-body imaging of an adult mouse at a uniform micrometer resolution with a light sheet microscope

A transparently embedded adult (6 weeks old) *Thy1-YFP-16* mouse whole-body sample was imaged with the ASI SPIM light sheet microscope in combination with the milling platform as described above. Images were sequentially acquired over two channels (488 nm and 568 nm). We achieved 46-Hz imaging for a 1.64 × 0.82 mm^2^ field of view. This configuration corresponds to a voxel resolution of 0.8 × 0.8 × 3.5 µm.^3^ Consequently, the collection of ~20 million two-channel images (2048 × 1024 pixels each) for all slices from the whole sample (100 × 35 × 20 mm^3^) required ~120 h of imaging time, which resulted in ~70 terabytes of data for the two channels.

This large quantity of data resulted in a challenge for data processing. We thus developed a custom software pipeline to implement automated volume stitching including linear channel unmixing, intra-slab stitching and inter-slab stitching. Linear channel unmixing substantially reduced the autofluorescence level in the final images, which has been a major issue for whole-body imaging.^18,19^ No transformation algorithm was needed.^17^

**Fig. 2:**
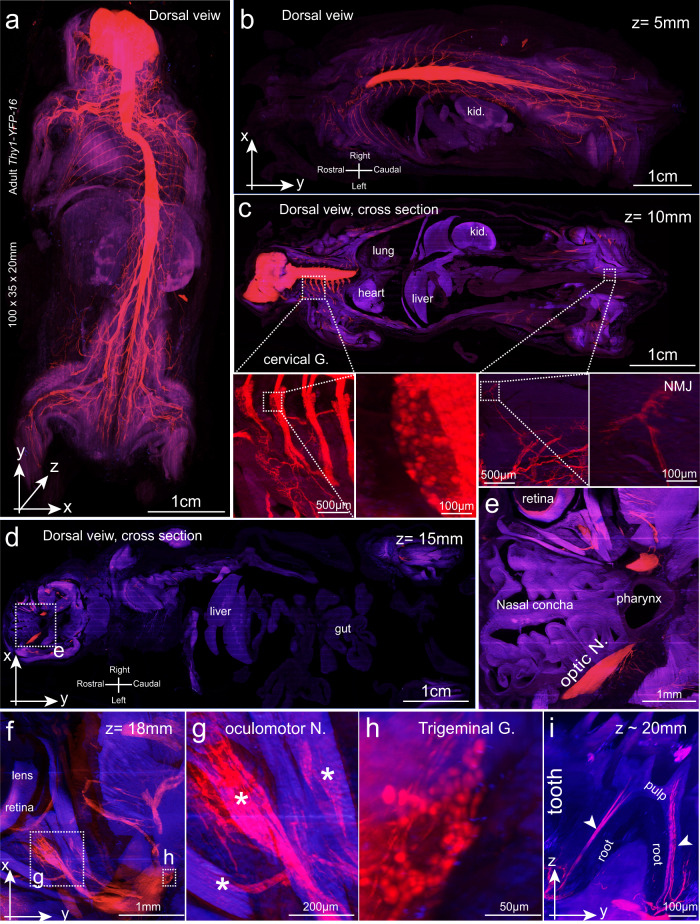
Whole-body imaging of a 6-week-old *Thy1-YFP-16* mouse at micrometer resolution with a light sheet microscope. Images were acquired with a 10×/0.3 NA objective on an ASI SPIM (voxel size, 0.8 × 0.8 × 3. µm^3^). Red, GFP signal; blue, autofluorescence. **a** 3-D image volume (as MIP) of the whole sample. **b**–**e** Optical blocks were acquired at a *z*-depth of 5 mm (**b**), 10 mm (**c**), and 15 mm (**d**). Boxed regions in **c** were enlarged to display cervical DRG (cervical G.), NMJ and internal organs including the kidney (kid.), liver, lung and heart. The boxed region in **d** was enlarged in **e** to display structures including the nasal concha, pharynx, optic nerve (optic N.) and retina. **f** An optical block acquired at a *z*-depth of 18 mm shows the eye structures, including the lens and extraocular muscles. **g**, **h** Boxed regions in **f** were enlarged to show the extraocular muscles (asterisks), oculomotor nerve (**g**) and trigeminal ganglion (**h**). **i** A *z*–*y* optical block at ~20 mm shows the innervation (arrowheads) of a mouse molar within the dental pulp and root.

Reconstructed images revealed the entire CNS and PNS throughout the whole body (Fig. 2a). Horizontal optical blocks (*x*–*y*) acquired at *z*-depths of 5 mm, 10 mm, 15 mm and 18 mm all displayed comparable resolutions and quality (Fig. 2b–d, g). Although no YFP signal was detected in the internal organs, autofluorescence clearly showed the fine organization of the internal organs including the liver, kidney, lung, gut and heart among others (Fig. 2b–d; Supplementary information, Fig. S4f). Head and neck structures are the most difficult to visualize in whole-body imaging studies.^2,5^ Here, the cervical spinal cord and ganglia, nasal concha, pharynx, optical nerves, retina, trigeminal ganglion neurons, extraocular muscles and oculomotor nerve were all clearly visualized (Fig. 2c, e–i). Because of the high transparency of hard-tissue samples, even nerves within a tooth were well displayed (Fig. 2I; Supplementary information, Fig. S4b and Video S1).

Optical blocks acquired in a sagittal (*y*–*z*) or coronal (*x*–*z*) orientations indicated image continuity after reconstruction and a uniform image resolution (Supplementary information, Fig. S4a–i). Dorsal root ganglion (DRG) neurons in all spinal cord segments could be visualized, including the head, cervical, thoracic, lumbar and sacral regions (Supplementary information, Fig. S4e–i and Video S1). Neuromuscular junctions (NMJs) could also be visualized throughout the whole body (Fig. 2c; Supplementary information, Fig. S4e3, f, g). The renal tubule and gut villi were also visible because of their autofluorescence (Supplementary information, Fig. S4h).

### Whole-body imaging of a mouse pup at micrometer resolution with a confocal microscope

Next, we tested whole-body imaging with a confocal microscope system. The body of a *Thy1-YFP-16* mouse pup at P5 was processed according to the immersion protocol for the body trunk. The brain and internal organs were removed because no signal was detected in the internal organs as expected for this mouse strain and the signal in the brain was too strong. All other tissues remained intact including the skin. The body trunk turned transparent after 2 weeks and remained equally transparent after transparent embedding (Supplementary information, Fig. S1b).

**Fig. 3:**
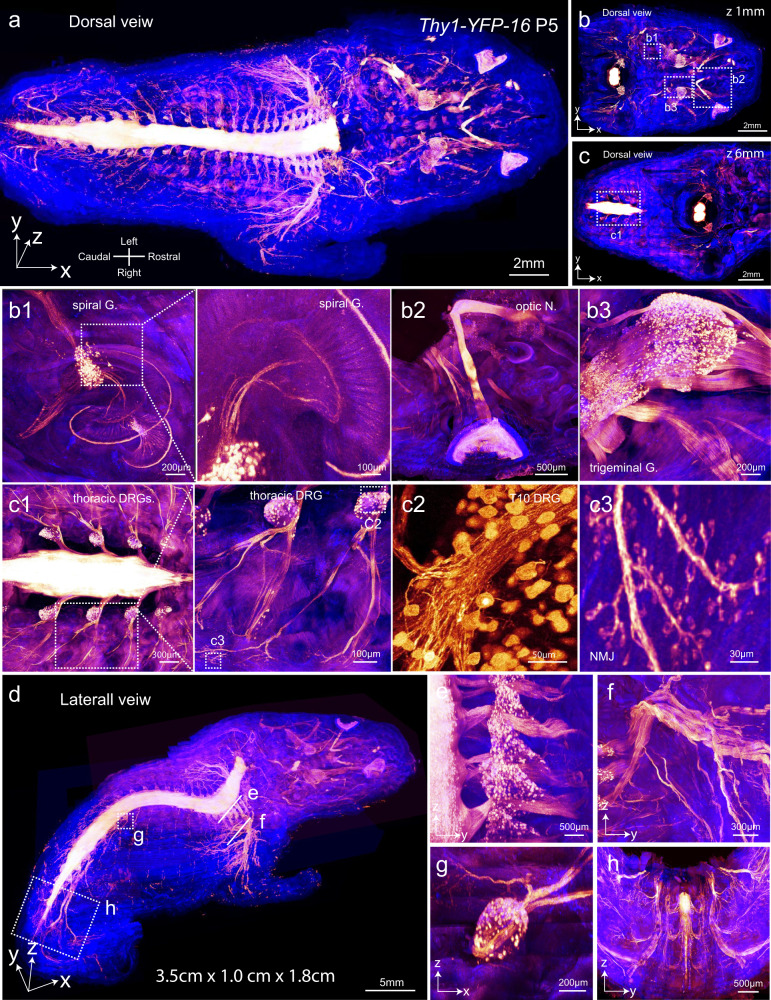
Whole-body imaging of a mouse pup at micrometer resolution with a confocal microscope. The body trunk of a *Thy1-YFP-16* mouse pup at P5 was processed following the TESOS protocol and imaged with a 20×/0.95 NA immersion objective (gold, YFP signal; blue, autofluorescence) (voxel size, 0.9 × 0.9 × 3.5 µm^3^). **a** The final image stack of 3.5 cm (*x*) × 1.0 cm (*y*) × 1.8 cm (*z*) was stitched from 40 slabs with a thickness of ~500 μm each. **b**, **c** Image in **a** was re-sliced at a *z*-depth of 1 mm (**b**) or 6 mm (**c**). Boxed regions were enlarged to show details. **b1** Spiral ganglion (spiral G.). Boxed region was enlarged to show axons innervating hair cells. **b2** Optic nerve and retina. **b3** Trigeminal ganglion (trigeminal G.). **c1** DRGs at the level of the thoracic vertebrae. Boxed region was enlarged to show individual DRG (**c2**) and NMJs (**c3**). **d**–**h** Lateral view of the image stack (**d**). Boxed regions were re-sliced to show the thoracic DRGs (**e**), branchial plexus (**f**), T10 DRG (**g**) and the sciatic nerve (**h**).

The sample was imaged with a Leica 20×/0.95 NA immersion objective on a confocal microscope combined with a stand-alone milling platform. The final image stack of 3.5 × 1.0 × 1.8 cm^3^ size was stitched from 40 stacks with a thickness of ~500 µm for each slab (Fig. 3; Supplementary information, Video S2). The voxel size was 0.9 × 0.9 × 3.5 µm^3^. Optical sections were acquired at various depths that showed a consistent resolution throughout the entire sample (Fig. 3b, c). At a *z*-depth of 1 mm, neuronal somas of the spiral ganglion were clearly visualized together with their axons innervating hair cells (Fig. 3b1). In addition, the retina, optic nerves, and trigeminal ganglia were also clearly displayed with somas and axon bundles (Fig. 3b2, b3). At a *z*-depth of 6 mm, the thoracic segment of the spinal cord was displayed. Enlarged images clearly showed the thoracic DRG neurons and NMJs (Fig. 3c, c1, c2, c3). To display the structural continuity in the *z* dimension, the image stack was displayed in a lateral view (Fig. 3d) and y–z or x–z optical slices were acquired. The *y*–*z* optical slices showed four cervical DRGs (Fig. 3e). The *y*–*z* or *x*–*z* slices also showed intact structures of the branchial plexus, T10 DRG and sciatic nerves (Fig. 3f–h).

Although the imaging speed of a confocal system is ~10-fold slower than that of a light sheet system, even with a resonant scanner, the resolution and image quality of the former were obviously much better, despite the similarity in voxel size between the two systems.

### Uniform sub-micron resolution imaging of tissue samples

We next explored whether a uniform sub-micron resolution could be achieved for a large tissue sample. We chose to use confocal laser scanning microscope because no commercially available light sheet microscopes for cleared tissue could achieve sub-micron resolution.

A *Thy1-EGFP-M* mouse brain sample (1 × 1 × 1.5 mm^3^) was processed and imaged with a 40×/1.3 NA objective (voxel size 0.26 × 0.26 × 1.2 µm^3^). The rotary microtome was used and the final image stack was stitched from eight slabs (Supplementary information, Fig. S5a and Video S3). Optical slices indicated that high-quality images were acquired at all *z*-depths (Supplementary information, Fig. S4b–f). Dendritic spines and boutons could be visualized at depths of 300 µm and 1200 µm, respectively (Supplementary information, Fig. S5b2, f1–f3). The *y*–*z* optical slices indicated that image continuity in the *z* dimension was well preserved after reconstruction. Axons and dendrites remained continuous and intact in the *z* dimension (Supplementary information, Fig. S5g, g1–g3).

**Fig. 4:**
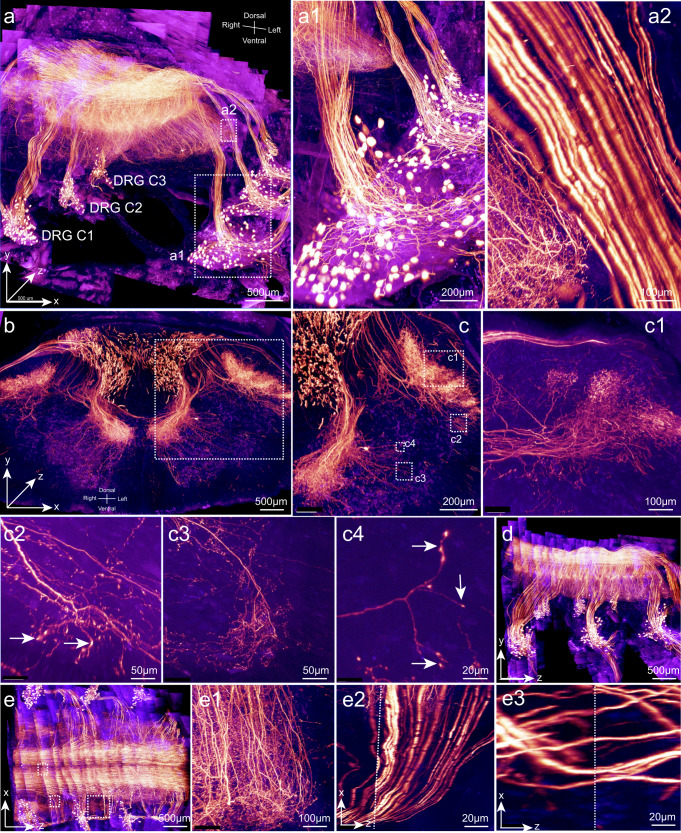
Sub-micron resolution imaging of DRG sensory neurons and their projections in the spinal cord. A segment of cervical vertebrae from an adult *Shh-Cre*^*ERT2*^*;Ai140* mouse, which contains the spinal cord, bones, attached muscle and skin (3.5 × 2.2 × 3 mm^3^) was processed and imaged with a 40×/1.3 NA oil immersion objective (voxel size, 0.37 × 0.37 × 1.2 µm^3^; gold, GFP signal; blue, autofluorescence). **a** Cervical DRG pairs from C1 to C3 were included in the reconstructed 3-D images. Boxed regions were enlarged in **a1** and **a2**. **b** A sub-block with a thickness of 500 µm is displayed to show the dense projections of DRG neurons in the spinal cord. **c** Boxed region in **b** was enlarged to show details. Boxed regions in **c** were enlarged and resliced in **c1**–**c4** to show axonal arbors in **c**. Axonal boutons were clearly visible (arrows in **c2** and **c4**). **d**, **e** A *y*–*z* view and *x*–*z* view of the image stack were displayed. Boxed regions in **e** were resliced and enlarged in **e1**–**e3**. Dotted lines in **e2** and **e3** indicated boundaries between 2 stitched adjacent stacks in the *z* dimension.

Sonic hedgehog (Shh)-genetic inducible fate mapping mice could be used to label sensory neurons within the DRGs.^20^ Here we induced *Shh-Cre*^*ERT2*^*;Ai140* mice at 6 weeks of age. The cervical body segment was transparently embedded and imaged with a 40×/1.3 NA objective on a confocal microscope (voxel size, 0.37 × 0.37 × 1.2 µm^3^) combined with a rotary microtome. We performed linear channel unmixing to distinguish autofluorescence from the GFP signal. The final image stack (3.5 × 2.2 × 3 mm^3^) was stitched from 22 slabs in the *z* dimension (Fig. 4a; Supplementary information, Video S4). Fine neural structures were clearly visualized, including the somas (Fig. 4a1), central axonal branches (Fig. 4a2), axonal arbors (Fig. 4b, c, c1) and boutons (Fig. 4c1–c4) of the DRG neurons. The image was continuous in the *z* dimension (Fig. 4d, e). Arbors and axons remain continuous in the *z* dimension (Fig. 4e1–e3).

Thus, the TESOS method is applicable to various tissue samples. It enabled uniform sub-micron resolution imaging of large samples, and the image continuity of neuronal axons and somas was well preserved after reconstruction.

### The TESOS method is compatible with immunofluorescence staining

To determine whether the TESOS method is compatible with immunofluorescence staining, we carried out whole-mount immunofluorescence staining of brain slices (1.5 × 1.5 × 1.0 mm^3^) from adult mice with antibodies against laminin.^4^ Samples were processed for transparent embedding and imaged with a 40×/1.3 NA objective on a confocal microscope (voxel size, 0.4 × 0.4 × 1.5 µm^3^) combined with a rotary microtome. The final image stack was stitched from 6 slabs in the *z* dimension (Supplementary information, Fig. S6a). Optical slices in the *x*–*y* orientation acquired at various depths showed comparable staining signals for the vasculature (Supplementary information, Fig. S6b, b1, c, c1, d, d1, e, e1). A sub-block in the *x*–*z* orientation showed continuous vasculature staining in the *z* dimension (Supplementary information, Fig. S6f). In addition, *x*–*z* optical slices also showed consistent vasculature staining signals and the reconstructed images remained continuous at all stitching boundaries (Supplementary information, Fig. S6g, g1–g6, h, h1, h2). We also performed double immunofluorescence staining for mouse brain slices with antibodies against laminin and glial fibrillary acidic protein. The vasculature and glial cells were clearly visible simultaneously (Supplementary information, Fig. S6i, i1–i3). Therefore, the TESOS method is compatible with immunofluorescence staining.

### Sub-micron resolution imaging of peripheral nerves within peripheral organs and the CNS

**Fig. 5:**
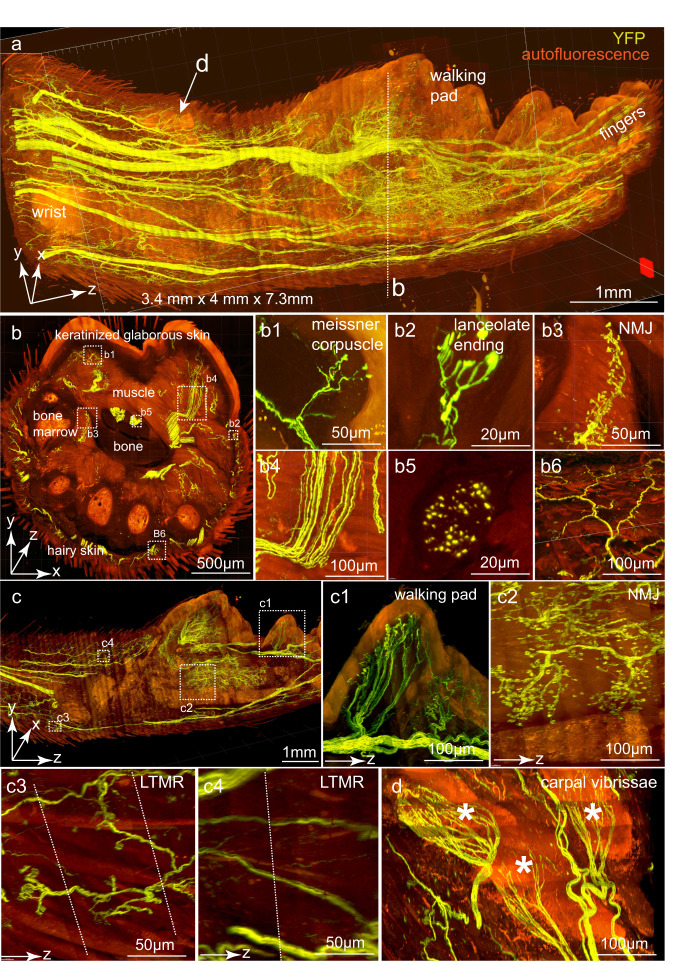
Sub-micron resolution imaging of peripheral nerve axons in an intact adult *Thy1-YFP16* mouse forepaw with skin and hair. An intact forepaw including the skin and hair from an adult *Thy1-YFP-16* mouse was imaged with a 40×/1.3 NA objective (voxel size, 0.4 × 0.4 × 1.2 µm^3^). The digits were not included in the image (yellow, YFP signal; red, autofluorescence). **a** Reconstructed final image stack of 3.4 (*x*) × 4.0 (*y*) × 7.3 (*z*) mm^3^ dimension was stitched from 35 slabs with a thickness of 250–300 µm thickness each. **b** A sub-block of 500 µm in thickness (i.e., the *z* dimension; dotted line in **a**) is shown in the *x*–*y* orientation. Boxed regions were enlarged and re-sliced to show various structures including a Meissner corpuscle (**b1**), lanceolate ending surrounding a hair follicle (**b2**), NMJs (**b3**), nerve bundles in a longitudinal orientation (**b4**), a nerve bundle cross-section (**b5**) and a sensory axon with its branches under the skin (**b6**). **c** A sub-block of 200 µm in the *x* dimension was shown in the *y*–*z* orientation to display the structure continuity in the *z* dimension. Boxed regions were enlarged and re-sliced to show various structures including sensory nerves within a walking pad (**c1**), NMJs (**c2**), LTMR innervating hair follicles (**c3**) and LTMR axons (**c4**). Dotted lines in **c3** and **c4** indicated boundaries between 2 stitched adjacent slabs in the *z* dimension. **d** Sensory nerves innervating the three carpal vibrissae (asterisks) at the wrist position (arrow in **a**).

We next explored whether the TESOS method can be used for tracing individual nerve axons in the PNS and CNS. The intact forepaw of an adult *Thy1-YFP-16* mouse (6 weeks old) was processed with all tissues preserved including hair, skin, muscle, bones and nerves (Supplementary information, Fig. S1a). The sample was imaged with a 40×/1.3 NA objective on a confocal microscope combined with a rotary microtome. The final image stack of 3.4 × 4.0 × 7.3 mm^3^ dimension was stitched from 35 slabs (Fig. 5a; Supplementary information, Video S5) (voxel size, 0.4 × 0.4 × 1.2 µm^3^). Linear channel unmixing was performed to improve the signal contrast. An *x*–*y* optical block was displayed (Supplementary information, Fig. 5b). Boxed regions were enlarged or re-sliced to display different sensory or motor nerve endings including Meissner corpuscles within the walking pad, lanceolate endings surrounding the hair follicle, NMJs, nerve axon bundles, nerve axons in cross-section and sensory nerve axon bundles under the skin (Fig. 5b1–b6). A *y*–*z* optical block showed the image continuity (Fig. 5c). Boxed regions were enlarged to display sensory nerves innervating a walking pad (Fig. 5c1), NMJs in the muscle (Fig. 5c2) and low-threshold mechanical receptor (LTMR) nerve axons under the hairy skin (Fig. 5c3, c4). Three carpal vibrissae located at the wrist of the forearm were also displayed (Fig. 5d).^21^

The sub-micron resolution enabled us to trace individual axons with Vaa3D.^22^ Nerve axons and endings were visualized within the glabrous skin of the walking pads (Supplementary information, Fig. S7a). All the axons under the walking pad are sensory axons that innervate Meissner corpuscles based on the mouse genotype, axons morphology and localization within the dermal papillae (Supplementary information, Fig. S7a1).^23^ All endings derived from individual axons could be traced (Supplementary information, Fig. S7b). We traced all axons within the five walking pads in this forepaw (Supplementary information, Fig. S7c, d). Around 5–15 sensory axons were labeled within each walking pad (Supplementary information, Fig. S7e1–e5). Each axon innervated 7.2 ± 3.14 Meissner corpuscles on average. The average area of the receptive field for each axon was 8620.00 ± 7820.80 µm^2^ (Supplementary information, Fig. S7f). The receptive fields between axons did not overlap (Supplementary information, Fig. S7g).

To trace nerve axons in the CNS*, hSyn-Cre* AAV was injected under the glabrous skin of the walking pads of *Ai140* mouse forepaws.^24^ Body segments corresponding to the cervical and thoracic vertebrae were collected. A transparently embedded sample (2.5 × 3.8 × 6 mm^3^) was imaged with a 40 × /1.3 NA objective under a confocal microscope (voxel size, 0.4 × 0.4 × 1.2 µm^3^) combined with a rotary microtome.

**Fig. 6:**
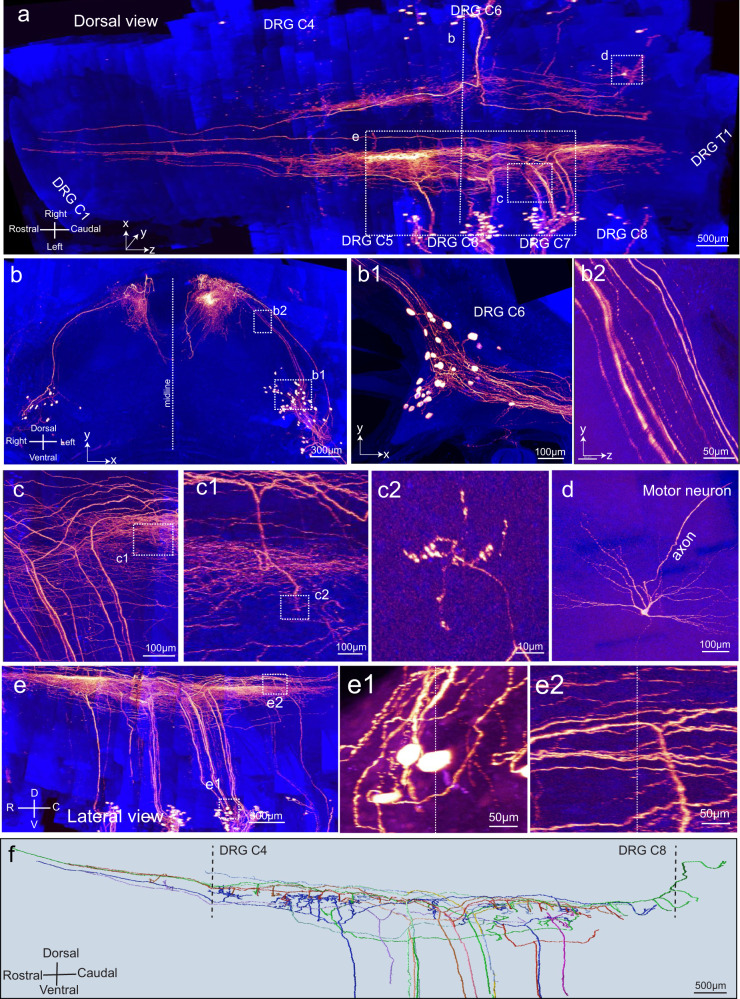
Sub-micron resolution imaging reveals complete projections of individual sensory neurons in the spinal cord. DRG neurons were labeled by injecting *Ai140* mouse pups (P6) with *Synapsin-Cre* AAV at the walking pad position. Cervical vertebrae were collected 2 months after injection and imaged with a 40×/1.3 NA objective (voxel size, 0.4 × 0.4 × 1.2 µm^3^; gold, GFP signal; blue, autofluorescence). **a** The final image stack of 2.5 × 3.8 × 6 mm^3^ dimension was constructed from 28 slabs with a thickness of 250–300 µm each. **b** Caudal view of the image stack. Boxed regions were enlarged and re-sliced to display details including DRG neurons (**b1,** **b2**). **c** Bifurcations of DRG neuron central branches. Boxed regions in **c** were enlarged to display an axonal arbor (**c1**) and boutons (**c2**). **d** A motor neuron that was randomly labeled. **e** Lateral view of the image stack. Boxed regions were enlarged to display DRG neurons (**e1**) and axons (**e2**). Dotted lines in **e1** and **e2** indicated boundaries between 2 stitched adjacent stacks in the *z* dimension. **f** Complete projection of 12 DRG neurons within the spinal cord.

The resulting uniform sub-micron resolution enabled us to identify the fine details of individual neurons within the spinal cord (Supplementary information, Fig. S8a) including the soma, peripheral and central axons, central axon bifurcation, axon endings, and the ten collateral branches together with their arbors (Supplementary information, Fig. S8b–d, d’, e1–e10). All labeled neurons were located from DRGs at C5–C8. Central projections were mostly in segments C4–C8 and were restricted to the medial side of the spinal cord (Fig. 6a, b; Supplementary information, Video S6). Individual DRG neurons and their central projection axons were clearly identified (Fig. 6b1, b2). In addition, axon collateral branches, arbors and boutons were clearly visualized (Fig. 6c, c1, c2). A sporadically labeled motor neuron was also visible (Fig. 6d). The structural continuity of axons and somas was not disrupted (Fig. 6e, e1, e2).

With Vaa3D, we were able to trace the complete spatial projection of axonal arbors and distinguish them from neighboring axonal arbors (Supplementary information, Fig. S9a–c). We outlined the complete central projection arbors of 12 DRG neurons (Fig. 6f; Supplementary information, Fig. S9). The findings are summarized below: (1) Most of the arbors are localized in DRGs between the C4 and T1 segments (10/12) (Supplementary information, Fig. S9d–i, k, l, n, o). No arbor was visualized rostrally beyond the C2 DRG. (2) The axonal arbors all projected to the medial side of the spinal cord in lamina 3, 4 or 5 (Supplementary information, Fig. S9d'–o'). (3) The collateral branches and arbors were not evenly distributed along the axons. Rostral branches could extend for 2–3 mm without collateral branches or arbors (Supplementary information, Fig. S9d, e, g, i–m). (4) No rostral branches extended beyond the C1 DRG into the brainstem. Most of them (9/12) ended as free terminals without arborization (Supplementary information, Fig. S9d, e, g, h–m). (5) Most caudal branches ended as arbors (10/12) (Supplementary information, Fig. S9d–m).

### Mesoscale projection mapping of sensory nerve axons from digits to the spinal cord at sub-micron resolution

**Fig. 7:**
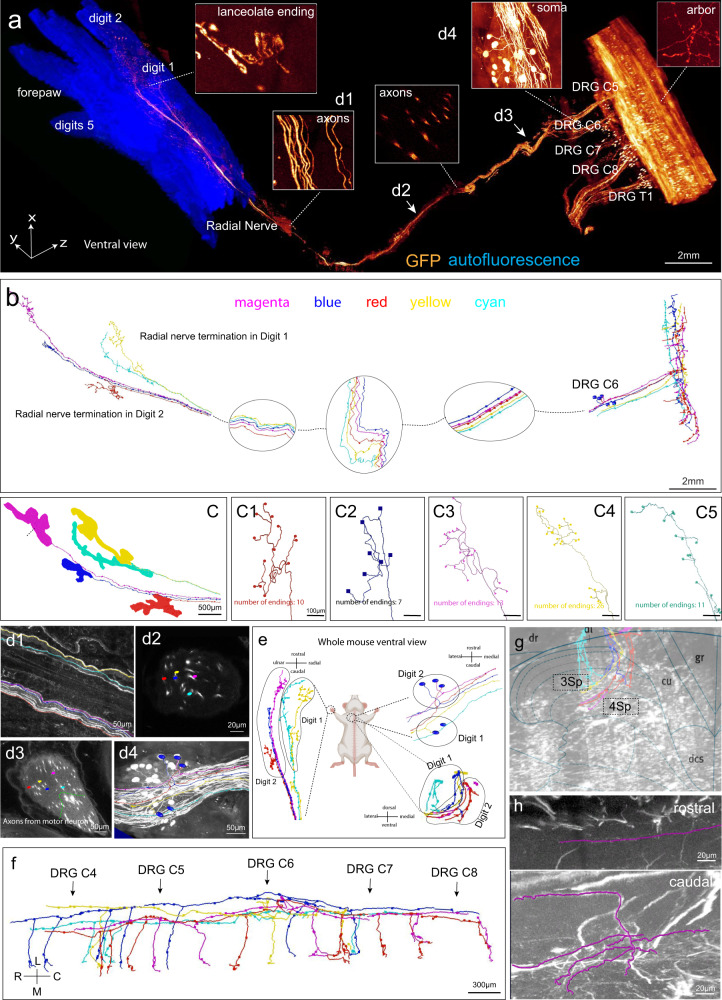
Mesoscale connectome mapping of individual sensory neurons from the digit hair follicles on the forepaw to the spinal cord. An adult *Thy1-EGFP-M* mouse was processed following the TESOS method. The embedded sample was selectively imaged with a 40 × /1.3 NA objective on a confocal microscope (voxel size, 0.4 × 0.4 × 1.5 µm^3^). **a** The final image stack (25 × 18 × 20 mm^3^) was stitched from 98 slabs with a thickness of 250–350 µm each. The indicated regions are enlarged in the boxes. **b** Complete tracing of 5 sensory neurons from hair follicles to the spinal cord with Vaa3D. Each color represents one neuron. **c** Receptive fields of the 5 sensory neurons innervating hair follicles on digits 1 and 2 (labeled with different colors). The terminals are shown as round dots in **c1**–**c5**. **d1**–**d4** Optical slices acquired at different positions along the radial nerve tracing course. Positions are indicated in **a**. **e** The topological relationship between the sensory receptive fields on the digits, neuron somas in the DRG and axonal projections in the spinal cord. **f** Complete tracing courses of the five neurons within the spinal cord. **g** Axonal projections were mapped to the Allen Spinal Cord Atlas at position C7. **h** The rostral (upper) and caudal endings (lower) of an axon.

We proceeded to test whether TESOS can produce projection mapping of individual peripheral nerve axons from PNS to CNS, which has never been achieved in the rodent model. An adult *Thy1-EGFP-M* mouse (6 weeks old) was processed for transparent embedding and segments from the forearm to cervical vertebra were selectively imaged with a 40×/1.3 NA objective on a confocal microscope (voxel size, 0.4 × 0.4 × 1.5 µm^3^) combined with a stand-alone milling platform. The final image stack (2.5 cm × 1.8 cm × 2 cm) included the entire forepaw, the radial nerve, part of the branchial plexus, cervical DRGs and the spinal cord from C2 to T2 (Fig. 7a; Supplementary information, Video S7). Axon tracing was performed with Vaa3D. We were ultimately able to reconstruct the complete projection courses of five DRG neurons. These entire projection courses extended ~4.5 cm from forepaw digits 1 and 2 to the DRGs and extended ~5 mm within the spinal cord (Fig. 7b).

The sub-micron resolution possible with this method enabled us to identify them as sensory neurons with lanceolate ending surrounding hair follicles in the digit (Fig. 7a). Their perceptive fields were positioned longitudinally along the digit axis (Fig. 7c). The numbers of lanceolate endings ranged from 7 to 26 (Fig. 7c1–c5). Their somas were located within the C6 DRG (Fig. 7d1–d4). The spatial relationships among the five axons were not constant within the radial nerve. The neuronal somas innervating digit 1 were localized more caudally than those of digit 2 (Fig. 7e). They projected to lamina 3 (3Sp) and lamina 4 (4Sp) in the spinal cord. Projection fields of digit 2 were localized more medially than those of digit 1 (Fig. 7g, e). In the rostral-caudal direction, projections of these neurons extended from the C3 DRG to the C8 DRG and each of them gave rise to 3–8 collateral branches (Fig. 7f). None of their rostral branches extended beyond C3. Each rostral branch ended as a free terminal, whereas caudal branches ended as arbors (Fig. 7h).

## Discussion

Tissue clearing technology was originally developed to acquire volumetric images of biological samples without sectioning. By rendering tissue transparent, internal 3-dimensional (3-D) organization of tissue could be directly visualized and reconstructed.^9^ Although many tissue clearing studies have demonstrated cellular structures throughout the whole body, the signal intensity and resolution were still inevitably lost when imaging deep regions of the sample. The signal loss could be attributed to at least two reasons. Firstly, no substance could achieve 100% transparency, which leads to signal intensity loss.^8^ Second, minor RI mismatch always occurs inside of the tissue, which leads to optical aberration and subsequent resolution loss.^25,26^

The TESOS method has provided a new strategy to solve the above issues. It is the combination of tissue clearing, transparent embedding, sectioning and block-face imaging together. Transparent embedding is the central concept of the TESOS method and both the “transparent” as well as the “embedding” aspects are essential. Formation of organogel within the tissue is the key to achieving both of them. In contrast to hydrogel,^3,7,27^ organogel contains no water and is composed of an organic solvent phase dispersed within a cross-linked polymer and has been used for drug delivery or cosmetic products.^28–30^ BED is a dental resin monomer known for its biosafety and strength.^31,32^ Both BB and BED have a high RI (BB, 1.56; BED, 1.55), which renders tissue highly transparent. Quadrol maintains the medium basic to protect GFP fluorescence.^4,33^ Irgacure 2959 was used to initiate the radical polymerization reactions. As compared with heat initiation, UV-initiation provides much better control over the reaction heat, which helps protect GFP activity. Overall, the whole clearing and UV-initiating polymerization processes are easy to manipulate and possess high reproducibility. Among the many recipes we have tested, transparent embedding was best achieved with BB-BED. In many other formulas, samples lost transparency after polymerization, despite their excellent transparency in the solution form. The BB-BED formulation is not compatible with any aqueous clearing method, but it can be adapted to other solvent-based clearing methods.

Both “transparent” and “embedding” are critical for the TESOS method on displaying its advantages over conventional tissue clearing or block-face imaging methods.

The “embedding” property enabled tissue samples to be sectioned without significant distortion. Transparent embedding is distinct from the agarose embedding used by many tissue-clearing studies for mounting or orienting samples.^5,17^ Agarose embedded samples from outside and was not dispersed within the tissue. The very high water content (>90%) made it too fragile to support mechanical sectioning stably.^17^ BB-BED medium was evenly distributed within the tissue and was directly converted by UV light into BB-poly BED resin to embed samples from the inside out. The strength of TESOS-embedded brain samples was over 100-fold stronger than the embedding agarose.

The “transparent” property distinguishes TESOS from section-reconstruction imaging strategies, like fMOST,^34^ and endows its flexibility on combining with various imaging systems, especially a light sheet microscope. The data acquisition rate of a light sheet microscope is 100-fold faster than point-scanning microscopes, which makes it the most optimal platform for imaging an adult mouse whole-body sample (100 × 35 × 20 mm^3^). Without “transparency”, this combination is impossible. Other block-face imaging strategies, Mouselight or fMOST, could not be combined with a light-sheet microscope due to insufficient sample transparency.^16,17,34,35^ ViSoR2 method was based on a light sheet microscope, but is not applicable for complex peripheral organs due to insufficient embedding support.^15,36^

Whole-body imaging based on conventional tissue clearing methods achieved satisfied image quality for superficial organs, but could hardly visualize deep regions, especially in the head and neck. Therefore, in our study, we highlighted head and neck structures, including tooth, nasal concha, pharynx, cranial nerves, DRGs and intercostal nerves, etc., which were imaged at a uniform micron-level resolution, represented by neuron soma and NMJs. These data showed that the block-face imaging strategy is better than conventional clearing methods on achieving uniform resolution throughout the whole-body sample.

Strong autofluorescence from the skin, muscles and bone still constitutes a challenge for whole-body or peripheral organs imaging.^37^ Immunostaining with a nanobody has been used to boost the fluorescence signal.^2^ In our study, because of the excellent preservation of endogenous fluorescence, we were able to use linear channel unmixing method to subtract autofluorescence from the GFP signal to improve both image quality and the SNR.^18,19^

Uniform sub-micron resolution imaging of large samples of over centimeters has never been achieved by conventional tissue clearing methods. We achieved this with confocal microscopes. Despite the slower imaging speed, the confocal microscope is more robust than the light sheet microscope in generating high-resolution, high-quality images. In addition, with transparent embedding and MagMount device, the TESOS method could be easily combined with various confocal microscopes without modification, showing great potential in performing the method in any regular lab.

Uniform sub-micron resolution imaging across large samples of diversified tissue revealed the spatial projection of individual peripheral neuronal axons within the PNS and CNS. The TESOS method revealed the spatial organization of sensory nerve axons and LTMRs under hairy and glabrous skin. Previously, these kinds of studies could only be performed using flat-mounted skin.^38,39^ Understanding the central organization and processing of the informational input from the sensory organs is a fundamental research topic for neuroscientists. In the direct dorsal column pathway model, individual LTMR neurons convey information directly into brainstem dorsal column nuclei (DCN), where the information is relayed.^40–42^ More recently, an LTMR-recipient zone (LTMR-RZ) model was proposed. In this model, hairy skin LTMR synapses reside mainly within the spinal cord dorsal zone with few synapses in the DCN, indicating the spinal cord interneurons as the relay center for integrating somatosensory input.^43–45^ Our tracing results of 12 DRG neurons provide direct evidence to support the hypothesis that sensory neurons for glabrous skin fit in the LTMR-RZ model. All the somas of the DRG neurons that were labeled by AAV after injection under the walking pad were localized in the C5–C8 DRGs. Their arbors were found mostly between C4 and T1 segments and did not extend into the brainstem.

Our study has provided the first full-course reconstruction of individual sensory neurons from digits to the spinal cord. A sensory neuron extends its axon from peripheral organs to the spinal cord with soma located in DRG, which makes projection mapping a tremendous challenge. Tissue clearing methods including vDISCO, wildDISCO and HYBRiD have successfully generated whole-body maps of the mouse nervous system.^2,5,46^ Yaron et al. have reconstructed partial-complete neuromuscular connectomes from newborn, developing and adult mice.^47^ However, none of the previous approaches had achieved complete single-neuron projection mapping from the PNS to CNS in a rodent model. vDISCO, wildDISCO and HYBRiD methods are limited by imaging resolution in the deep regions. In this study, we reconstructed five sensory neurons innervating hairy skin LTMRs from forepaw digits to the spinal cord, which covers a distance of >4.5 cm. *Thy1-EGFP-M* strain was chosen because of its strong GFP fluorescence and relatively sparse neuron labeling. Although tracing results from five neurons were insufficient for drawing substantial conclusions, they do underscore the usefulness of the TESOS method as a new tool for high-resolution connectome mapping in both the CNS and PNS.

The TESOS method also has its own limitations. Sectioning operation increases the entire operation time and makes re-imaging samples impossible. In addition, dehydration treatment led to uneven tissue shrinkage among different tissue types, which may lead to unexpected tissue deformation in some regions. Future studies may work on modifications of TESOS formulation to further improve the mechanical strength of polymerized samples in order to achieve better sectioning or milling performance. Furthermore, an innovative customized automated “imaging-milling” system might help save operation time and effort, especially for large samples.

## Materials and methods

### Animals

Mice were purchased from the Jackson Lab with genotypes including *C57BL/6* (JAX # 000664), *Thy1-EGFP-M* (JAX# 007788), *Thy1-YFP-16 *(JAX #003709), *Ai14* (JAX# 007908), *Ai 140* (JAX# 030220), *Shh-Cre*^*ERT2*^ (JAX# 005623) and *Gli1-cre*^*ERT2*^ (JAX# 007913). All animal experiments were approved by the Institutional Animal Care and Use Committee of the Chinese Institute for Brain Research.

For tamoxifen treatment, tamoxifen (Sigma-Aldrich, T5648) was dissolved in corn oil (Sigma-Aldrich, C8267) at 20 mg/mL. The solution was kept at –20 °C and delivered via intraperitoneal injection or oral gavage for postnatal treatments.

### Preparation of TESOS solutions

#### Decalcification medium

Decalcification solution was composed of 20% (w/v) Ethylenediaminetetraacetic acid disodium salt dihydrate (EDTA-Na_2_, Sigma-Aldrich, E5134) in water. Sodium hydroxide (Sigma-Aldrich, S5881) was used for adjusting the pH value to 7.0–7.5.

#### Melanin bleaching solution

Formula of melanin bleaching solution was modified based on previous literature.^48^ Bleaching solution A: 1% H_2_O_2_ (v/v) (Sigma-Aldrich, H1009), 15% urea (w/v) (Sigma-Aldrich, U5378) in 0.05 M Tris (Sigma-Aldrich, T6066), pH 10.0. Bleaching solution B: 3% H_2_O_2_ (v/v), 15% urea (w/v) in 0.05 M Tris, pH 10.0.

#### Decolorization medium

Quadrol medium (Sigma-Aldrich, 122262) was diluted with H_2_O to a final concentration of 25% (w/v). Due to the very vicious property of Quadrol, weighing is more convenient than measuring the volume. Quadrol medium can be warmed in a 45 °C water bath to increase flowability.

#### Gradient tB delipidation medium

tB (Sigma-Aldrich, 360538) was diluted with water to prepare gradient delipidation solutions: 30% (v/v), 50% (v/v) and 70% (v/v). 5% (w/v) Quadrol (Sigma-Aldrich, 122262) was added to adjust the pH over 9.5.

#### tB-Q dehydration medium

Dehydrating medium was composed of 70% (v/v) tB and 30% (w/v) Quadrol.

#### BB-BED clearing medium (RI 1.552)

BB-BED clearing medium is composed of 47% (v/v) benzyl benzoate (BB) (Sigma-Aldrich, W213802), 48% (v/v) of BED (Sigma-Aldrich, 413550), 5% (w/v) Quadrol and then supplemented with 2% w/v of Irgacure 2959 (2-Hydroxy-4’-(2-hydroxyethoxy)−2-methylpropiophenone, Sigma-Aldrich, 410896) as the UV initiator. The BB-BED medium is a colorless to slightly yellowish liquid (RI, 1.552) and can be preserved at 4 °C in the dark.

#### BB-PEG objective immersion medium

BB-PEG medium was used as the objective immersion medium for transparently embedded samples. BB-PEG is the clearing solution of our previous PEGASOS method and is composed of 75% (v/v) BB, 25% (v/v) PEG MMA500 (Sigma-Aldrich, 447943) and supplemented with 5% (w/v) Quadrol.^4^ It is compatible with all brands of oil- or water-immersion objectives. The medium can be dropped on the sample surface without a coverslip, which increases the working distance by 170 µm.

### Perfusion and tissue preparation

For trans-cardiac perfusion, mice were anesthetized with an intraperitoneal injection of a combination of xylazine and ketamine anesthetics (Xylazine 10–12.5 mg/kg; Ketamine, 80–100 mg/kg body weight). Ice-cold heparin PBS of 50–100 mL (10 U/mL heparin sodium in 0.01 M PBS) was injected transcardially to flush the blood. 50 mL 4% PFA (Sigma-Aldrich, P6148) in 0.01 M PBS, pH 7.4) was then injected transcardially for fixation.

### Skin viral labeling

Skin viral injection protocol was modified based on previous publication.^24,38^ *Ai140* (Jax# 030220) reporter mouse pups of P6 were used for viral injection. High titer (>10^12^ GC/mL) AAV2/1-hSyn-Cre-WPREhGH virus (Addgene, 105553-AAV1) was diluted 1:8 or 1:4 in 0.9% saline and 1 µL was injected under the forepaw skin. Mice were euthanized 2 months after injection.

### Quantification of relative Young’s modulus

For quantification of Young’s modulus, a column with a diameter of 3.5 mm and height of 10 mm was formed with 3% agarose, wax, and BB-BED solvent, respectively. Fixed brain and cleared brain were cut into the same shape for the test. Young’s modulus was measured with a tabletop Uniaxial Testing Machine (TestResources, USA), with compression load at the speed of 0.02 mm/s. The average Young’s modulus of a fixed brain was normalized as 1.00, and the ratio of Young’s modulus of other tissue or materials to that of a fixed brain was calculated as the “relative Young’s modulus”.

### TESOS clearing procedure through passive immersion

A detailed step-by-step protocol of the TESOS method is provided in the Supplementary information, Data S1. For samples containing hard tissues, 4% PFA fixation was performed at room temperature for 24 h and then samples were decalcified in 20% EDTA-Na_2_ (pH 7.0) at 37 °C on a shaker for 4 days. Samples were then washed with distilled water for at least 30 min to remove excessive EDTA. Samples were next decolorized with the Quadrol decolorization solution for 2 days at 37 °C on a shaker. Samples were placed in gradient tB delipidation solutions for 1–2 days and then tB-Q for 2 days for dehydration. Finally, samples were immersed in the BB-BED medium in a shaker at room temperature for at least 1 day until transparency was achieved.

For soft tissue organs, decalcification treatment was skipped. After 24 h fixation, samples were treated with Quadrol decolorization solution on a shaker for 2 days at 37 °C. Samples were next treated with gradient delipidation solutions in shaker at room temperature for 1–2 days, followed by tB-Q dehydration treatment for 1–2 days. Finally, samples were placed in the BB-BED clearing medium in a shaker at room temperature for at least 1 day until final transparency was achieved.

For body trunk sample of large size, processing time for each step can be elongated.

The time schedule for clearing different types of tissue with the immersion method is summarized (Supplementary information, Table S1).

After clearing, samples could be preserved in the BB-BED medium at 4 °C in the dark.

### Clearing of adult mouse whole-body samples through active recirculation

In our whole-body clearing procedure, adult mice (6 weeks old) of BL6 background with black hair and pigmented skin were used. After anesthesia, mouse hair was shaved with an electrical clipper followed by cosmetic depilatory cream application following the manufacturer’s guide. The depilatory cream and hair were removed by wiping the skin with a moistened cloth. After perfusion and overnight fixation with 4% PFA, the sample was wrapped with a paper towel wetted with melanin bleaching solution A, placed in a sealed plastic container and shaken (100 rpm) at 37 °C for 6 h. The bleaching solution A was refreshed every 2 h. Next, the sample was fully immersed in the melanin bleaching solution B and shaken (100 rpm) at 37 °C for 2 h. The pigment was mostly enriched in the mice’s back skin and absent on the abdomen. After the melanin bleaching step, the entire skin should present pale white. After melanin bleaching, the sample was washed with 0.05 M Tris (pH 10.0) solution for 20 min 3 times followed by overnight wash in the same solution.

Afterward, various solutions were pumped into the left ventricle using a recirculation setup described previously.^4^ In brief, the decolorization solution was recirculated into the sample at 37 °C for 2 days with daily solution change. Next, the decalcification solution was recirculated at 37 °C for one week with daily solution change. 50% tB and 70% tB were recirculated at room temperature for 3 days and 4 days sequentially. tB-Q dehydration solution was recirculated for 3 days at room temperature. BB-BED clearing solution was recirculated for one week until final transparency was achieved. Air bubbles within the sample tissue could be removed by vacuum treatment for 1 h. Totally, one month was needed for clearing an adult mouse whole-body sample.

### Quantification of fluorescence intensity

For quantification of fluorescence and autofluorescence intensity changes of whole-body melanin bleaching treatment in Supplementary information, Fig. S1f, the fixed *Thy1-YFP-16* mouse samples were used. Melanin bleaching treatment was performed following the above protocol. Fluorescent images of back skin and subcutaneous muscle were taken before and after treatment with a Zeiss stereo fluorescence microscope with the same zoom factor and exposure time. The “integrated intensity” values were measured using Image J (NIH). The average signal intensity of samples before treatment was defined as initial intensity and was normalized as 1.0. The ratio of “integrated intensity” value after treatment to initial intensity was calculated to evaluate the fluorescence intensity change.

Half brain slices of 0.5 mm thickness from adult *Thy1-EGFP-M* mice were used for quantification of GFP fluorescence intensity change in Supplementary information, Fig. S2a. Intestine slices (2 × 2 × 0.5 mm^3^) from adult *Gli1-cre*^*ERT2*^*; Ai14* after Tamoxifen induction for 2 weeks were used for quantification of tdTomato fluorescence intensity change in Supplementary information, Fig. S2b. Fluorescent images of each time point were taken with a Zeiss stereo fluorescence microscope with the same zoom factor and exposure time. Fluorescence intensity change was calculated as stated above. The average signal intensity of samples after fixation was defined as initial intensity and was normalized as 1.00.

Half brain samples from adult *Thy1-EGFP-M* mice were used for quantification of GFP fluorescence intensity before and after polymerization in supplementary Fig. S2e. Images were acquired with Leica confocal microscope using a 20×/0.95 NA objective at various depths before and after polymerization. Fluorescence intensity change was calculated as stated above. Average signal intensity at the depth of 500 µm before polymerization was defined as the initial intensity and was normalized as 1.00.

### Quantification of SNR

Half brain samples from adult *Thy1-EGFP-M* mice were used for imaging and quantification of SNR in Supplementary information, Fig. S2f. Images were acquired with Leica confocal microscope using a 20×/0.95 NA objective at various depths.

To quantify the SNR of “TESOS (after embedding)”, we imaged the embedded sample from the surface to a certain *z*-depth without sectioning. To quantify the SNR of “TESOS (after sectioning)”, we set the imaging depth as 500 µm from the surface for each slab, and section off 400 µm before imaging the next slab. The same position was found and imaged as “TESOS (after embedding)”.

The SNR was calculated by:

$${SNR}=\frac{\mu }{\sigma }=\frac{S-{I}_{{background}}}{\sqrt{S-{I}_{{background}}+{\sigma }_{{background}}^{2}}}$$

where *S* is the average signal intensity value in the image, *I*_background_ and *σ*_background_ are the background’s mean and standard deviation (SD), respectively.^49^

### Transparent embedding of cleared samples through UV-initiated polymerization

The transparent embedding could be performed as early as 48 h after the sample transparency was achieved. For small samples (<10 mm), disposable base molds of various sizes (VWR M-475) were used. Samples were placed in the mold filled with BB-BED medium and covered with a coverslip. Make sure that the sample is ~5 mm away from the side wall. The mold was placed on ice and irradiated with a high-power UV curing light (Thorlabs CS20K2) with typical power of 50 mW/cm^2^ for a cleared mouse brain sample. The lamp head was ~10 cm from the sample. Curing duration was 5–10 min. Alternatively, inexpensive industrial UV point light source from eBay or Taobao could be used. The power setup and curing time need to be optimized. At any time, the sample temperature should not be over 50 °C for over 10 min to preserve endogenous fluorescence. After UV curing, the sample block was preserved in a 50 mL tubes with ~3–5 mL BB-BED medium in it. For large samples including mouse pup whole body or adult mouse body trunks, customized glass chambers were used. The UV curing was performed from all angles with the duration being determined empirically. Typically, a mouse’s whole body could be polymerized within 30 min.

Transparently embedded samples remained clear as in the solvent solution. The transparency could reduce 7 days after curing, possibly due to oxidation and uneven polymerization inside the tissue.

### Imaging of transparently embedded samples using a confocal/2P microscope combined with a rotary microtome

#### Confocal/2P microscope setup

An upright Leica Sp8 or Nikon AX confocal microscope was used for acquiring confocal imaging data. A resonant scanner (8000 Hz) was equipped on both models. Leica Sp8 was equipped with a piezo sample stage (Leica) on top of a long-range stage (Scientifica MMBP). Nikon AX was equipped with an ASI piezo *z*-stage on top of a long-range stage (ASI FTP2000). A Upright Zeiss 880 2 P microscope was used for acquiring two-photon images.

#### Sample mounting using the MagMount setup

A magnetic kinematic base (KB25/M or SB1/M, Thorlabs) was used for mounting and transferring samples. The kinematic base was designed for re-mounting optical parts precisely with high repeatability and is composed of two magnetically connected plates (top, bottom). Embedded samples were glued onto the top plate with the epoxy resin (Taobao or Gorilla, Home Depot).

A MagMount setup was installed onto the sample movement stage under the microscope, which is composed of a bottom plate screwed onto a 2-axis goniometer stage (Thorlabs GNL20/M). Another bottom plate was installed on a rotary microtome (Sakura ACCU-CUT SRM or Leica 2050 in our lab) through a mounting base (Thorlabs BA1/M) (Supplementary information, Fig. S2a).

Prior to imaging a sample, alignment was performed to ensure the sectioning plane is parallel to the imaging plane. Alignment is composed of two steps. The first step is rough adjustment. A top plate with no sample was connected onto the microtome bottom plate. The top plate surface was visually examined and adjusted using the microtome adjustment handles till it is parallel to the microtome movement plane during sectioning process. The rough adjustment was performed by assuming that both microscope imaging plane and microtome sectioning plane are roughly parallel or perpendicular to the ground level.

In the second step, fine alignment was performed through the MagMount. A top plate with a test sample was sectioned on the microtome to expose a large area of sample surface. The sectioned sample was transferred to the MagMount under the microscope and the top surface was scanned with a 10× objective. A large tile scan will reveal which regions of the sample surface is below the imaging plane. The goniometer was then adjusted and the surface was scanned again until all regions of the sample surface appear in the surface scanning simultaneously.

The alignment is only needed at the beginning of an imaging process using a rotary microtome setup.

#### Sample imaging and sectioning

Following alignment, embedded samples were mounted on the microtome and sectioned (5 µm/cut) to expose the sample’s top surface. After sectioning, the surface was dropped with a BB-PEG immersion medium and imaged with an upright confocal/2P microscopy as a regular slide. Oil immersion objectives are highly recommended. The imaging depth of each imaging *z*-stack is determined mainly by the objective’s working distance. The transparently embedded sample can be imaged without a coverslip. Therefore, a 40×/1.3 NA oil immersion objective could reach 410 µm imaging depth (240 µm + 170 µm) on the sample. The top plane of the *z*-stack should be at least 10 µm below the sample sectioning surface to avoid any potential distortion on the machined surface.

After imaging the selected sample areas, the sample was transferred to the microtome for sectioning (5–10 µm/round). The sectioning depth should be at least 10% less than the *z*-stack depth to provide an overlapping area for stack stitching. The sectioned sample was transferred back to the microscopy stage and dropped with BB-PEG immersion medium for the next imaging cycle (Fig. 1e).

### Imaging of an adult mouse whole-body sample using a light sheet microscope and a milling platform

A whole-body imaging platform was built based on the ASI ct-dSPIM **(**ASI Dual selective plane illumination microscope for cleared tissue) (Supplementary information, Fig. S2b). The microscope was equipped with two lasers of wavelengths 488 nm and 561 nm (both Coherent). Both illuminating and imaging objectives (10×X/0.3 NA Olympus) were positioned at 45^°^ to the sample surface. Images were collected with a CMOS camera (PRIME BSI). Dual-color imaging was acquired by sequential imaging of individual channels. Long-range stages (ASI FTP2000) were used for *x*-, *y*- and *z-*axis movement. A customized silicone chamber filled with BB-PEG medium was fixed on the stage platform. During imaging, the *x*-stage moved smoothly at a speed of 1.75 mm/s. The *y*-step size was set to provide ~10% overlap between adjacent image strips.

The long-range *xyz* stage was installed on a linear translational platform (OMTOOLS EPSA-200) to transfer the sample between imaging and milling positions. The milling motor (Huanyang 300 W 60 K RPM) equipped with a 6 mm flat-end diamond milling bur (Taobao) was installed on a vertical translational stage (OMTOOLS EPSA100) standing next to the microscope. The device was controlled by custom software written in Python based on the programmable interfaces (APIs) provided by the device manufacturers.

### Workstation and HPC computing platform

Confocal image data was stored and processed in a workstation equipped with dual Intel Platinum 8252c CPU, an NVIDIA RTX Titan graphic card and 512 GB of memory. It was equipped with a disk array consisting of 4× 8TB SSDs configured in RAID5, connected via 10 GB/s fiberoptic intranet. Windows 10 Pro 64-bit and Linux dual-operating systems were run on the workstation.

Whole-body image data was stored and processed in the HPC computing platform. The HPC Platform contains a total of 3312 processors, with 60 compute nodes, 13 fat nodes and 6 GPU nodes. The cluster’s total double precision computing capability is 371TFlops. A 4.2 Petabyte (PB) bare high-performance storage system with a capacity of up to 40 GB/s real-time aggregated read/write bandwidth was used for large-scale data transfer and storage.

### Linear channel unmixing for autofluorescence reduction

For tissues with strong autofluorescence including skin, skeletal muscles, bones, and etc., linear channel unmixing was performed to distinguish signal from autofluorescence. Autofluorescence signal widely spreads from 350 nm to 600 nm,^50^ whereas fluorescence from protein or antibody conjugation is restricted. Depending on the endogenous fluorescence spectrum, the autofluorescence detection channel was set up at either 488 nm or 568 nm wavelength. The channel unmixing operation was performed with the “image calculator” plugin in Image J (NIH Image J). The autofluorescence signal was subtracted from the signal channel.

### Confocal image data reconstruction

Confocal images were acquired and stored in .lif or .nd2 formats depending on different platforms. Deconvolution was accomplished with the microvolution module within the Slidebook 6.0 software (3I inc.). The iteration number was set as 10. Regularization was set as “none”. Blind deconvolution was checked. PSF model was automatically generated based on the optical parameter being provided.

Intra-slab stitching was accomplished with BigStitcher plug-in within the Image J. 10% overlapping was set up for adjacent tiles. Alternatively, built-in stitching modules in LAS X (Leica) or NIS Element (Nikon) could also be used. After intra-slab stitching, images were exported as image sequences in .tif format for Inter-slab stitching.

Inter-slab stitching was performed sequentially based on the overlapping regions. Pairwise stitching plug-in of the Image J could be used. Alternatively, custom software written in Python could be used.

### Automated reconstruction of light sheet microscope images

Light sheet images were acquired as OME-TIFF first, and then converted into .tiff images. We used the lossless Lempel-Ziv-Welch (LZW) algorithm to compress the raw images and reached a compression ratio of 2:1.

#### Intraslab stitching

Adjacent strips were selected for stitching. Due to the very large data size (~100 GB/strip), the two strips were down-sampled into multiple hierarchies and the number of hierarchies was automatically determined based on the data size. The stitching operation was started from the bottom level hierarchy. The two strips were shifted in *x*, *y*, *z* dimensions until the normalized cross-correlation (NCC)^51^ of overlapping regions achieved highest value. The translation values in *x*, *y* and *z* were scaled up to the higher-level hierarchy. The adjustment was performed again based on the NCC value. In this way, the 3-D shift was determined roughly in the lower-level hierarchy of small data size and refined in the higher hierarchy until the top hierarchy of raw data size was adjusted. In this way, the computation time could be greatly reduced. After intraslab translations of all adjacent strips were calculated, the new positions were recalculated by minimum spanning tree (MST) algorithm and saved in a txt file for subsequent interslab stitching.

#### Interslab stitching

The two adjacent slabs were down-sampled into multiple hierarchies. The number of hierarchies was automatically determined by software according to the data size. One slice from a slab was selected and compared with all other slices from the other slab for their similarity. Similarity analysis was first performed in the low hierarchy. The pairwise feature points of the slice images were calculated and selected by random sample consensus (RANSAC).^51^ The two images were aligned by randomly selected pairwise points, and the NCC values of overlapping images were calculated. The translation was determined by a set of pairwise feature points that maximized NCC. For the following higher hierarchies, the NCC values were calculated while two images were shifted in a smaller range, until the most precise translation was determined in the top hierarchy. After calculating translations of all pairwise planes, the two planes which had the highest NCC value were considered overlapping planes. The new positions were saved for subsequent image output and analysis.

### Visualization

For 3-D rendering of most image datasets, image sequences were converted into .ims format (Imaris Converter, Bitplane). 3-D rendering, snapshots and animation were performed with Imaris (Bitplane).

To generate video of whole-body sample, image data was down-sampled by ratio of 3(*x*) x 3(*y*) x 2(*z*) and then converted into .nim format. 3-D rendering, snapshots and animation were performed with Atlas. (https://atlas-doc.readthedocs.io/en/latest/help.html).

### Axon tracing

A multiresolution pyramid was generated from stitched image sequences for 3-D tracing.^22^ Manual tracing of axons was performed by a team of two annotators using TeraFly in Vaa3D.^22,52^ For registration, spinal cord image stacks were re-sliced for planes orthogonal to the rostro-caudal axis of the spinal cord. The re-sliced imaging plane was aligned and annotated based on the spatial map for the Allen Mouse Spinal Cord Atlases (the Allen Institute).

### Statistic and reproducibility

*n* numbers are reported in figures and legends. Data are presented as mean ± SD using one-way ANOVA or Student’s *t*-test. Statistical analysis was performed in GraphPad Prism and Microsoft Excel.

### Supplementary information

Supplementary information, Video S1

Supplementary information, Video S2

Supplementary information, Video S3

Supplementary information, Video S4

Supplementary information, Video S5

Supplementary information, Video S6

Supplementary information, Video S7

Supplementary information, Figure S1

Supplementary information, Figure S2

Supplementary information, Figure S3

Supplementary information, Figure S4

Supplementary information, Figure S5

Supplementary information, Figure S6

Supplementary information, Figure S7

Supplementary information, Figure S8

Supplementary information, Figure S9

Supplementary information, Data S1

Supplementary information, Table S1

Supplementary information, video legend

## Data Availability

The linear channel unmixing pipeline, the codes for whole-body image stitching and the codes for confocal microscope image data stitching were deposited in our GitHub repository (https://github.com/Albertd1ng/TESOS_stitcher).

## References

[1] Tainaka K, Kuno A, Kubota SI, Murakami T, Ueda HR (2016). Chemical principles in tissue clearing and staining protocols for whole-body cell profiling. Annu. Rev. Cell Dev. Biol..

[2] Cai R (2019). Panoptic imaging of transparent mice reveals whole-body neuronal projections and skull-meninges connections. Nat. Neurosci..

[3] Chung K (2013). Structural and molecular interrogation of intact biological systems. Nature.

[4] Jing D (2018). Tissue clearing of both hard and soft tissue organs with the PEGASOS method. Cell Res..

[5] Nudell V (2022). HYBRiD: hydrogel-reinforced DISCO for clearing mammalian bodies. Nat. Methods.

[6] Tainaka K (2014). Whole-body imaging with single-cell resolution by tissue decolorization. Cell.

[7] Treweek JB (2015). Whole-body tissue stabilization and selective extractions via tissue-hydrogel hybrids for high-resolution intact circuit mapping and phenotyping. Nat. Protoc..

[8] Ueda HR (2020). Whole-brain profiling of cells and circuits in mammals by tissue clearing and light-sheet microscopy. Neuron.

[9] Ueda HR (2020). Tissue clearing and its applications in neuroscience. Nat. Rev. Neurosci..

[10] Roth RH, Ding JB (2020). From neurons to cognition: technologies for precise recording of neural activity underlying behavior. BME Front..

[11] Chen, Y. et al. A versatile tiling light sheet microscope for imaging of cleared tissues. *Cell Rep*. **33**, 108349 (2020). 10.1016/j.celrep.2020.108349 33147464

[12] Gao L (2015). Optimization of the excitation light sheet in selective plane illumination microscopy. Biomed. Opt. Express.

[13] Zeng H (2018). Mesoscale connectomics. Curr. Opin. Neurobiol..

[14] Winnubst J (2019). Reconstruction of 1,000 projection neurons reveals new cell types and organization of long-range connectivity in the mouse brain. Cell.

[15] Xu F (2021). High-throughput mapping of a whole rhesus monkey brain at micrometer resolution. Nat. Biotechnol..

[16] Li A (2010). Micro-optical sectioning tomography to obtain a high-resolution atlas of the mouse brain. Science.

[17] Economo MN (2016). A platform for brain-wide imaging and reconstruction of individual neurons. Elife.

[18] McRae TD, Oleksyn D, Miller J, Gao YR (2019). Robust blind spectral unmixing for fluorescence microscopy using unsupervised learning. PLoS One.

[19] Chorvat D (2005). Spectral unmixing of flavin autofluorescence components in cardiac myocytes. Biophys. J..

[20] Brownell I, Guevara E, Bai CB, Loomis CA, Joyner AL (2011). Nerve-derived sonic hedgehog defines a niche for hair follicle stem cells capable of becoming epidermal stem cells. Cell Stem Cell.

[21] Niederschuh SJ, Witte H, Schmidt M (2015). The role of vibrissal sensing in forelimb position control during travelling locomotion in the rat (*Rattus norvegicus*, Rodentia). Zoology.

[22] Peng H, Ruan Z, Long F, Simpson JH, Myers EW (2010). V3D enables real-time 3D visualization and quantitative analysis of large-scale biological image data sets. Nat. Biotechnol..

[23] Taylor-Clark TE (2015). Thy1.2 YFP-16 transgenic mouse labels a subset of large-diameter sensory neurons that lack TRPV1 expression. PLoS One.

[24] Bloom DC, Watson ZL, Neumann DM (2019). Peripheral AAV injection for retrograde transduction of dorsal root and trigeminal ganglia. Methods Mol. Biol..

[25] Masson A (2015). High-resolution in-depth imaging of optically cleared thick samples using an adaptive SPIM. Sci. Rep..

[26] Ye H, Shi G (2022). High-resolution multiscale imaging enabled by hybrid open-top light-sheet microscopy. BME Front..

[27] Yang B (2014). Single-cell phenotyping within transparent intact tissue through whole-body clearing. Cell.

[28] Skilling KJ (2014). Insights into low molecular mass organic gelators: a focus on drug delivery and tissue engineering applications. Soft Matter.

[29] Yuan T (2019). The ultrafast assembly of a dipeptide supramolecular organogel and its phase transition from gel to crystal. Angew. Chem. Int. Ed. Engl..

[30] Tan T (2020). Self-assembly of pentapeptides in ethanol to develop organogels. Soft Matter.

[31] Xie XJ (2017). Novel rechargeable calcium phosphate nanoparticle-containing orthodontic cement. Int. J. Oral Sci..

[32] Fleisch AF, Sheffield PE, Chinn C, Edelstein BL, Landrigan PJ (2010). Bisphenol A and related compounds in dental materials. Pediatrics.

[33] Schwarz MK (2015). Fluorescent-protein stabilization and high-resolution imaging of cleared, intact mouse brains. PLoS One.

[34] Zheng T (2013). Visualization of brain circuits using two-photon fluorescence micro-optical sectioning tomography. Opt. Express.

[35] Tao C, Xia C, Chen X, Zhou ZH, Bi G (2012). Ultrastructural analysis of neuronal synapses using state-of-the-art nano-imaging techniques. Neurosci. Bull..

[36] Wang H (2019). Scalable volumetric imaging for ultrahigh-speed brain mapping at synaptic resolution. Natl. Sci. Rev..

[37] Monici M (2005). Cell and tissue autofluorescence research and diagnostic applications. Biotechnol. Annu. Rev..

[38] Kuehn ED, Meltzer S, Abraira VE, Ho CY, Ginty DD (2019). Tiling and somatotopic alignment of mammalian low-threshold mechanoreceptors. Proc. Natl. Acad. Sci. USA.

[39] Neubarth NL (2020). Meissner corpuscles and their spatially intermingled afferents underlie gentle touch perception. Science.

[40] Mountcastle VB (1957). Modality and topographic properties of single neurons of cat’s somatic sensory cortex. J. Neurophysiol..

[41] Niu J (2013). Modality-based organization of ascending somatosensory axons in the direct dorsal column pathway. J. Neurosci..

[42] Johnson KO (2001). The roles and functions of cutaneous mechanoreceptors. Curr. Opin. Neurobiol..

[43] Abraira VE (2017). The cellular and synaptic architecture of the mechanosensory dorsal horn. Cell.

[44] Bai L (2015). Genetic identification of an expansive mechanoreceptor sensitive to skin stroking. Cell.

[45] Li L (2011). The functional organization of cutaneous low-threshold mechanosensory neurons. Cell.

[46] Mai, H. et al. Whole-body cellular mapping in mouse using standard IgG antibodies. *Nat. Biotechnol*. 10.1038/s41587-023-01846-0 (2023). 10.1038/s41587-023-01846-0 PMC11021200 37430076

[47] Meirovitch, Y. et al. Neuromuscular connectomes across development reveal synaptic ordering rules. *bioRxiv*10.1101/2021.09.20.460480 (2021).

[48] Chung JY (2016). A melanin-bleaching methodology for molecular and histopathological analysis of formalin-fixed paraffin-embedded tissue. Lab. Invest..

[49] Zhao Y (2022). Isotropic super-resolution light-sheet microscopy of dynamic intracellular structures at subsecond timescales. Nat. Methods.

[50] Zipfel WR (2003). Live tissue intrinsic emission microscopy using multiphoton-excited native fluorescence and second harmonic generation. Proc. Natl. Acad. Sci. USA.

[51] Emmenlauer M (2009). XuvTools: free, fast and reliable stitching of large 3D datasets. J. Microsc..

[52] Peng H, Bria A, Zhou Z, Iannello G, Long F (2014). Extensible visualization and analysis for multidimensional images using Vaa3D. Nat. Protoc..

