# Supplementary material for: Mapping of individual sensory nerve axons from digits to spinal cord with the transparent embedding solvent system
Source: Cell Res. 2024 Jan 3;34(2):124–39. doi: 10.1038/s41422-023-00867-3 (PMC10837210; doi:10.1038/s41422-023-00867-3)
Supplement: Supplementary file 8 — Supplementary information, Figure S1 [file 41422_2023_867_MOESM8_ESM.docx]

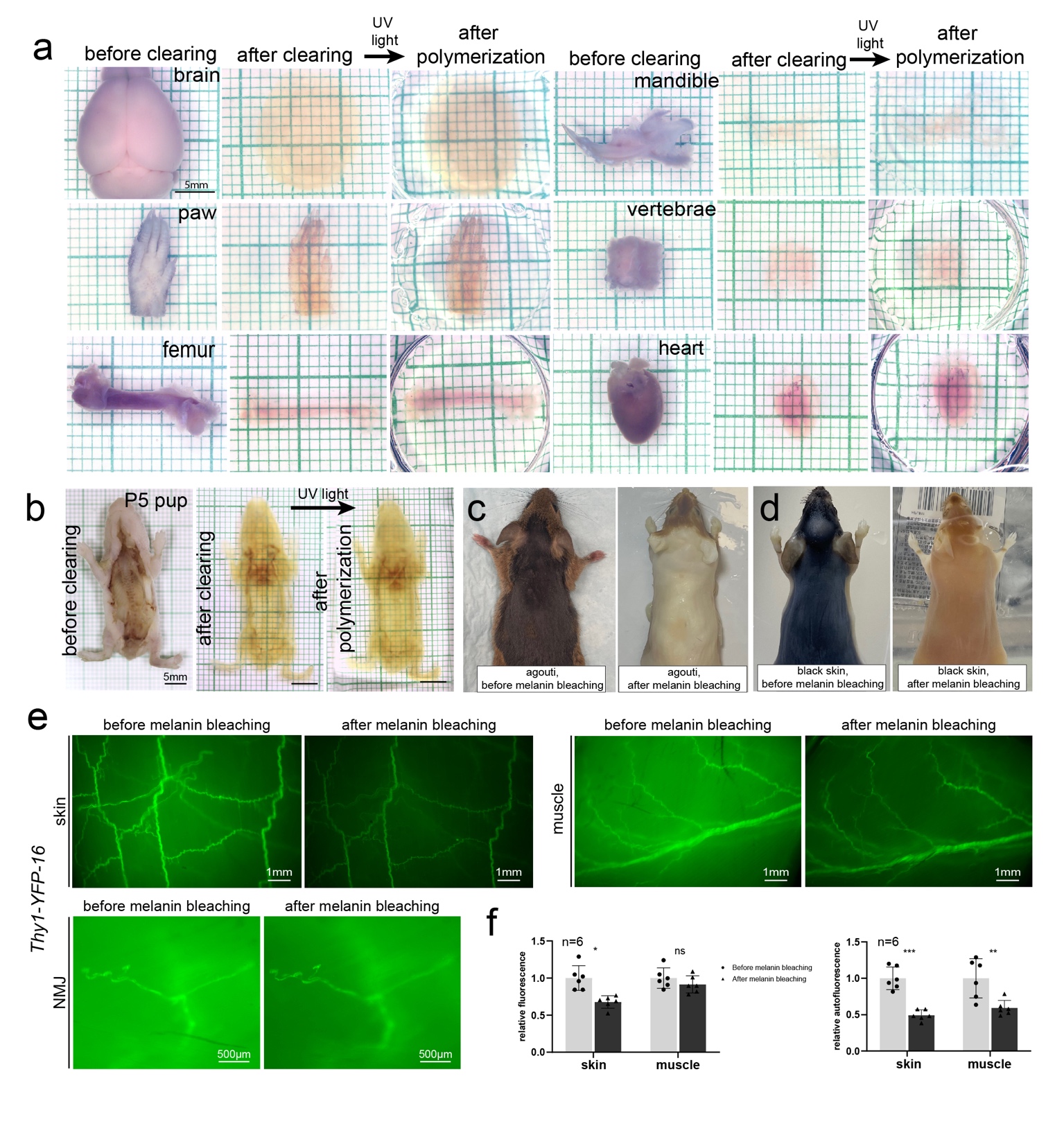


**Figure S1. The TESOS method enables transparent embedding of various tissue and organs**.

(a). Representative images of a mouse brain, mandible, paw, vertebrae, femur and heart processed by a passive immersion clearing protocol before clearing, after clearing and after polymerization.

(b). The body trunk of a mouse pup at P5 was processed following the passive immersion protocol and imaged before clearing, after clearing and after polymerization.

(c). Adult agouti mouse was imaged before and after melanin bleaching.

(d). Adult black skin mouse was imaged before and after melanin bleaching.

(e). Impact of melanin bleaching treatment on nerve signals in *Thy1-YFP-16* mouse skin and muscle (*n*=6). *: *P* < 0.05; **: *P* < 0.01; ***: *P* < 0.001; ns: not significant..
