# Supplementary material for: Mapping of individual sensory nerve axons from digits to spinal cord with the transparent embedding solvent system
Source: Cell Res. 2024 Jan 3;34(2):124–39. doi: 10.1038/s41422-023-00867-3 (PMC10837210; doi:10.1038/s41422-023-00867-3)
Supplement: Supplementary file 9 — Supplementary information, Figure S2 [file 41422_2023_867_MOESM9_ESM.docx]

**
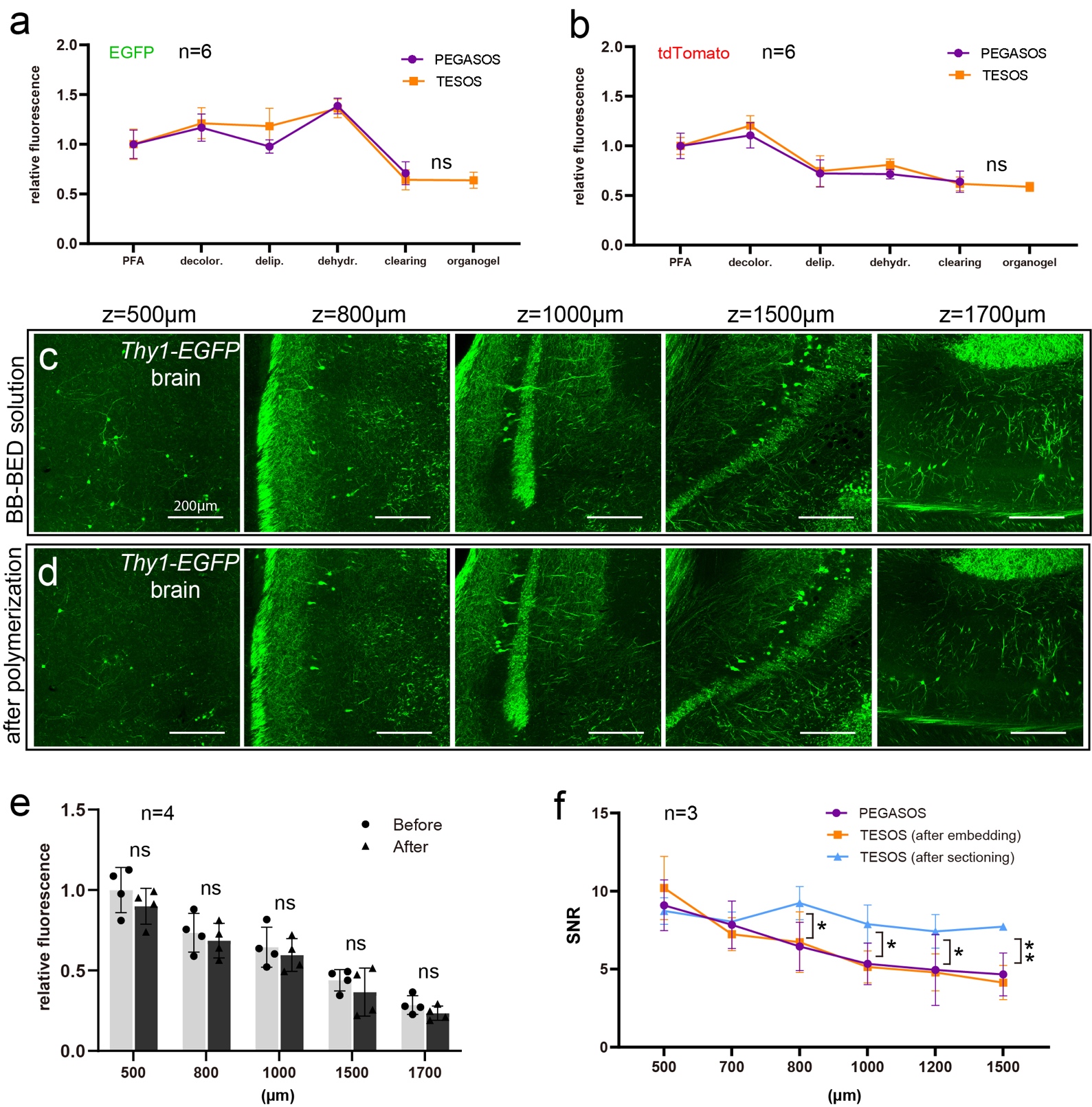
**

**Figure S2. TESOS treatment preserves endogenous fluorescence and does not** **compromise sample transparency.**

(a). Impact of TESOS treatment upon GFP fluorescence in comparison with the PEGASOS tissue clearing method. *Thy1-EGFP* mouse brain samples (*n*=6) were used. ns: not significant.

(b). Impact of TESOS treatment upon tdTomato fluorescence in comparison with the PEGASOS tissue clearing method. *Gli1-cre^ERT2^; Ai14* mouse intestine samples (*n*=6) were used. ns: not significant.

(c, d). *Thy1-EGFP* mouse brain was processed with the TESOS method. Images were acquired with a 20×/0.95 NA objective at various depths before (c) and after (d) polymerization.

(e). Quantitative analysis of relative fluorescence intensity before and after polymerization at various depths (*n*=4). ns: not significant.

(f). Samples were treated following the PEGASOS or TESOS protocol (*n*=3). Images were acquired in PEGASOS medium (purple), after polymerization (orange), or after sectioning (blue). SNR was calculated at various depths. Student’s *t* test was done between SNR after polymerization and SNR after sectioning at each z-depth. *: *P* < 0.05; **: *P* < 0.01.
