# Supplementary material for: Mapping of individual sensory nerve axons from digits to spinal cord with the transparent embedding solvent system
Source: Cell Res. 2024 Jan 3;34(2):124–39. doi: 10.1038/s41422-023-00867-3 (PMC10837210; doi:10.1038/s41422-023-00867-3)
Supplement: Supplementary file 10 — Supplementary information, Figure S3 [file 41422_2023_867_MOESM10_ESM.docx]

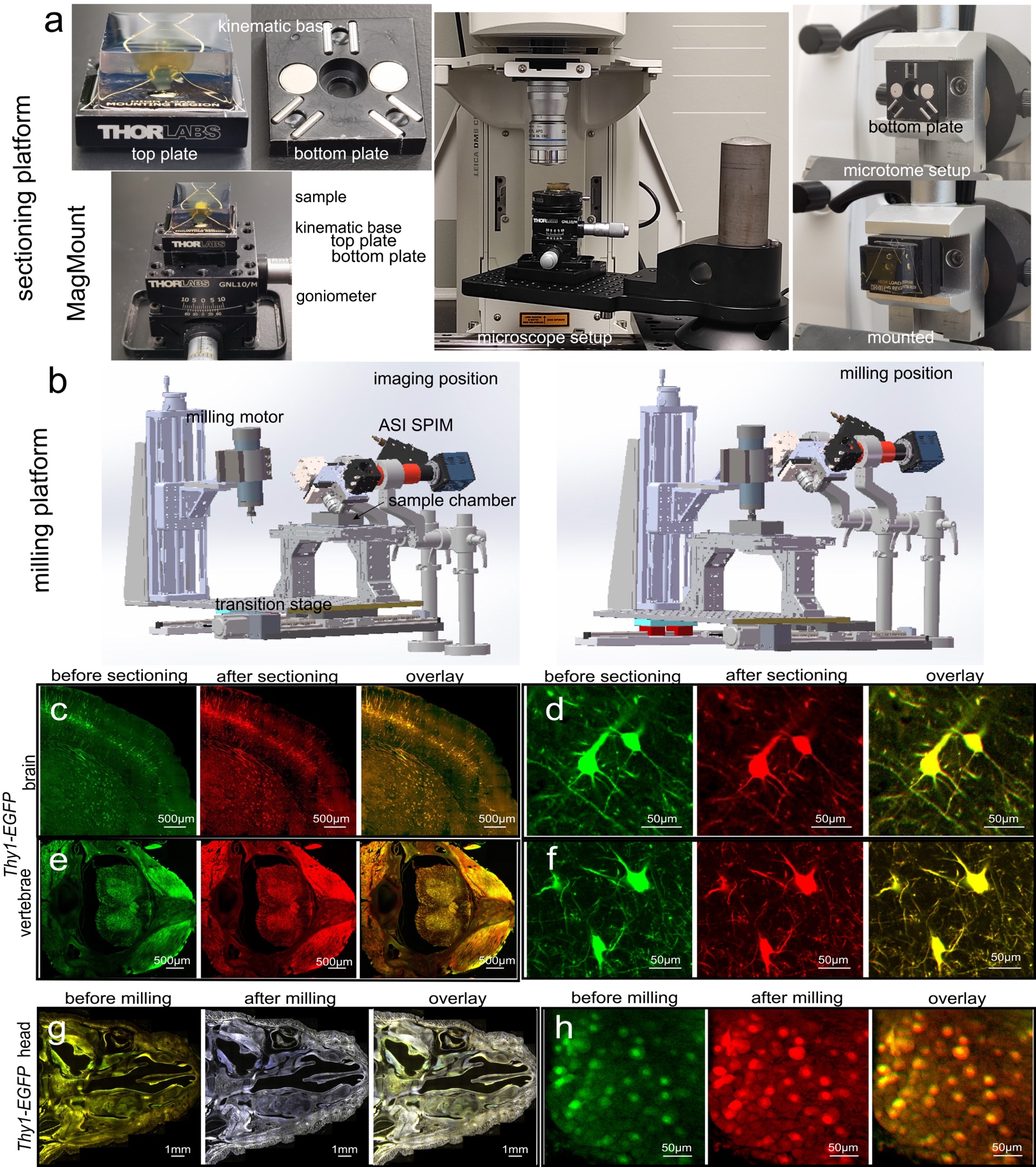


**Figure S3. Sectioning and milling platforms for processing transparently embedded samples.**

(a). The MagMount setup was designed for small samples and consists of a kinematic magnetic base and a two-axis goniometer. An embedded sample was glued onto the top-plate of the kinematic base. Another kinematic base bottom plate was secured on the rotary microtome. Kinematic base plates enable the mounted samples to be repositioned precisely.

(b). A milling platform was built next to the ASI SPIM for processing whole-body samples. A motorized linear stage moved the sample between the microscope and milling motor.

The brain of an adult *Thy1-EGFP* mouse was processed and embedded following the TESOS method for soft tissue organs. The vertebrae, femur and the head samples were all harvested from adult mice and embedded following the TESOS method for hard tissue organs. Images were acquired with a 10×/0.4 NA air or 20×/0.95 NA immersion objective before and after tissue was sectioned/milled from the sample. Imaging planes before sectioning/milling were 400µm below the surface. Samples of 350 µm in thickness were sectioned or milled off.

(c). The embedded brain was imaged before and after sectioning with the 10× objective. The two images were overlaid to show any changes in morphology. (d). Selected neurons were re-imaged with the 20× objective.

(e). The embedded vertebrae sample was imaged with the 10× objective before and after sectioning, and the resulting images were overlaid as in (c). (f). Selected neurons within the spinal cord were imaged with the 20X objective to show the absence of changes in neuronal morphology after sectioning.

(g). The head sample was imaged with the 10× objective before and after milling, and the resulting images were overlaid as in (c). (h). Selected ganglion neurons were re-imaged with the 20× objective to display consistent morphology.
