# Supplementary material for: Mapping of individual sensory nerve axons from digits to spinal cord with the transparent embedding solvent system
Source: Cell Res. 2024 Jan 3;34(2):124–39. doi: 10.1038/s41422-023-00867-3 (PMC10837210; doi:10.1038/s41422-023-00867-3)
Supplement: Supplementary file 11 — Supplementary information, Figure S4 [file 41422_2023_867_MOESM11_ESM.docx]

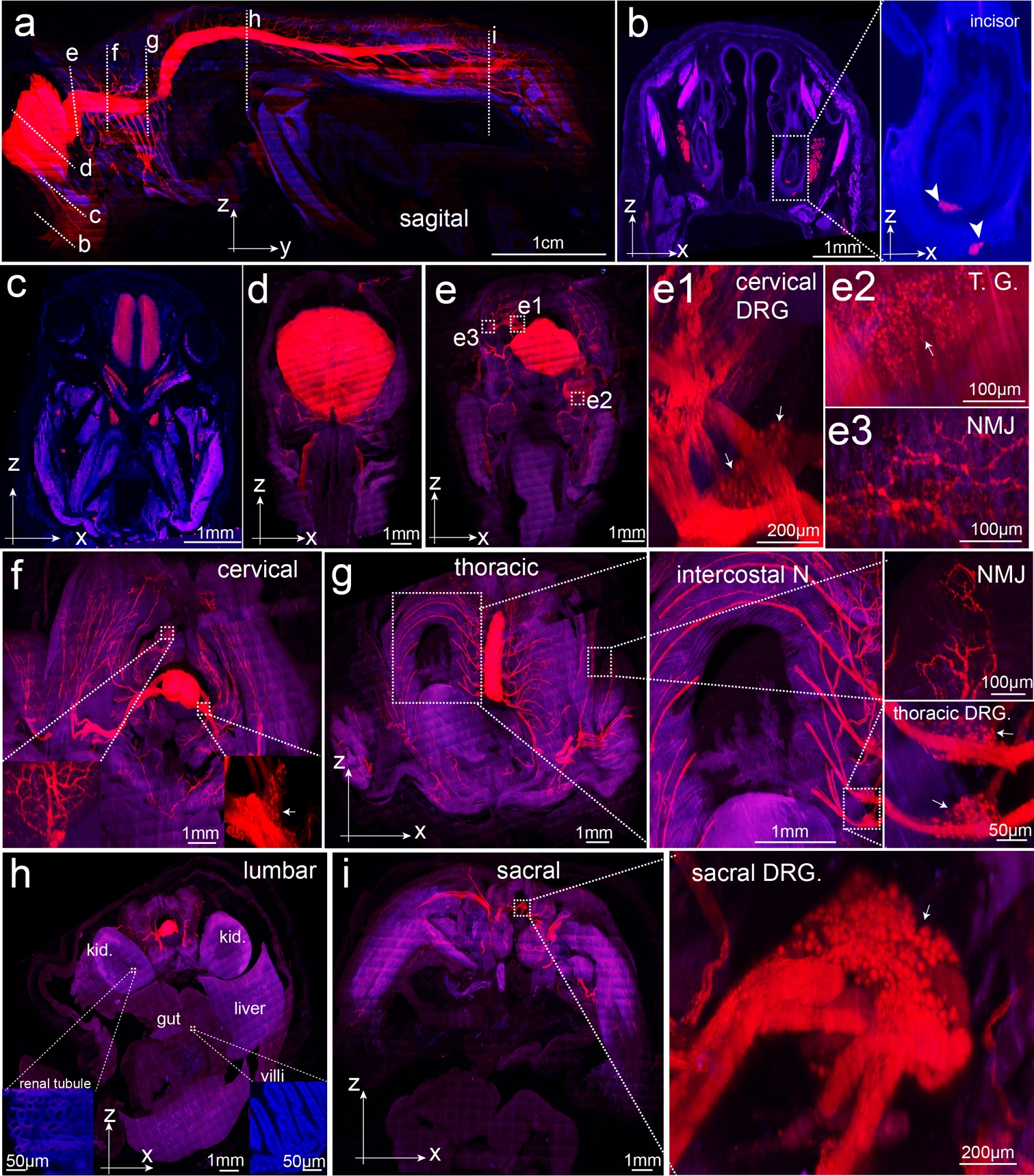


**Figure S4. Optical blocks from whole-body imaging of an adult *Thy1-YFP-16* mouse**.

(a). A sagittal optical block in the y-z dimension was acquired. Dotted lines indicate positions of the x-z optical blocks shown in the remaining pannels.

(b). Optical block acquired at the level of the nostrils. The enlarged region shows an incisor and innervating nerves (arrowheads).

(c). An x-z optical block at the level of the eye.

(d). An x-z optical block at the level of the brain.

(e). An x-z optical block at the level of the basal skull. Boxed regions were enlarged to display cervical DRG neurons (e1), the trigeminal ganglion (T.G. in e2) and neuromuscular junctions (NMJ, e3). Arrows show DRG neuron cell bodies.

(f). A coronal optical block at the cervical region. Boxed region was enlarged at corners to show DRG (right) and neuronal axons (left). Arrow shows DRG neuron cell bodies.

(g). An optical block at thoracic region. Boxed regions were enlarged to show intercostal nerves, NMJ and thoracic DRG neurons. Arrows show DRG neuron cell bodies.

(h). An optical block near the lumbar region. Kidney (kid.), gut and liver are shown. Two regions were re-sampled from the indicated positions to show the renal tubule and gut villi.

(i). An optical block from the sacral region. The boxed region was enlarged to show sacral DRG neurons. Arrows show DRG neuron cell bodies.
