# Supplementary material for: Mapping of individual sensory nerve axons from digits to spinal cord with the transparent embedding solvent system
Source: Cell Res. 2024 Jan 3;34(2):124–39. doi: 10.1038/s41422-023-00867-3 (PMC10837210; doi:10.1038/s41422-023-00867-3)
Supplement: Supplementary file 12 — Supplementary information, Figure S5 [file 41422_2023_867_MOESM12_ESM.docx]

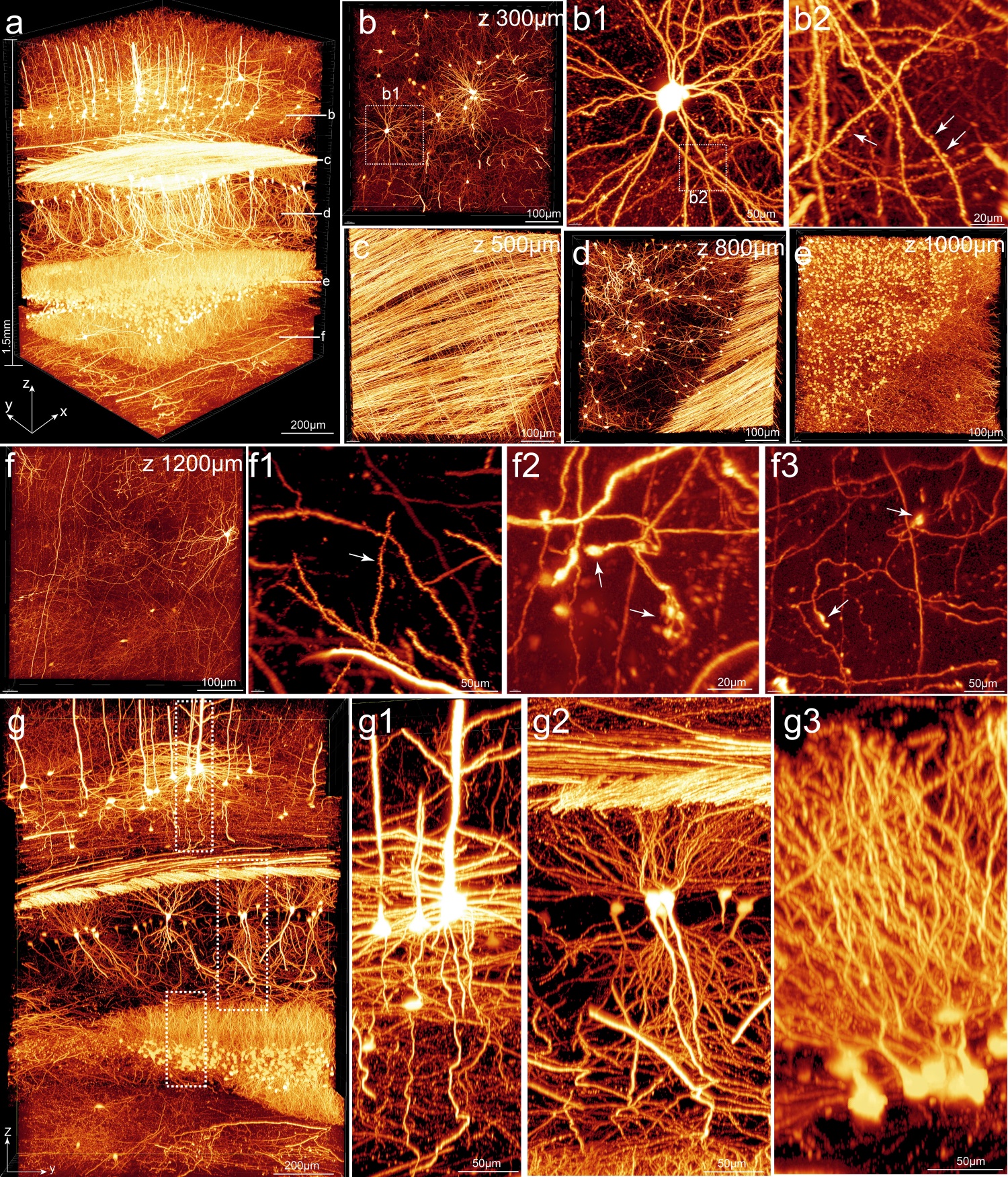


**Figure S5. Sub-micron resolution imaging of a *Thy1-EGFP* mouse brain sample**. A brain sample from an adult *Thy1-EGFP* mouse, which measured 1 mm (x) × 1 mm (y) × 1.5 mm (z) was processed and imaged with a 40×/1.3 NA objective (voxel size, 0.26 µm × 0.26 µm × 1.2 µm).

(a) The final image stack was stitched from 8 slabs, each with a thickness of 240 µm.

(b-f). Sub-blocks of 1 mm (x) × 1 mm (y) × 0.2 mm (z) were acquired at the z-depths indicated by the white lines in (a) and are displayed in the x-y orientation. The boxed region in (b) was enlarged in (b1). The boxed region in (b1) was resliced and enlarged in (b2) to display dendritic spines (arrows). Regions in (f) were resliced and enlarged to display dendritic spines (arrow in f1) and boutons (arrows in f2 and f3).

(g). A sub-block of 0.2 mm (x) × 1 mm (y) × 1.5 mm (z) was displayed in the y-z orientation. Boxed regions were enlarged to display descending neurons (g1), pyramidal neurons (g2) and granule cells (g3) respectively.
