# Supplementary material for: Mapping of individual sensory nerve axons from digits to spinal cord with the transparent embedding solvent system
Source: Cell Res. 2024 Jan 3;34(2):124–39. doi: 10.1038/s41422-023-00867-3 (PMC10837210; doi:10.1038/s41422-023-00867-3)
Supplement: Supplementary file 13 — Supplementary information, Figure S6 [file 41422_2023_867_MOESM13_ESM.docx]

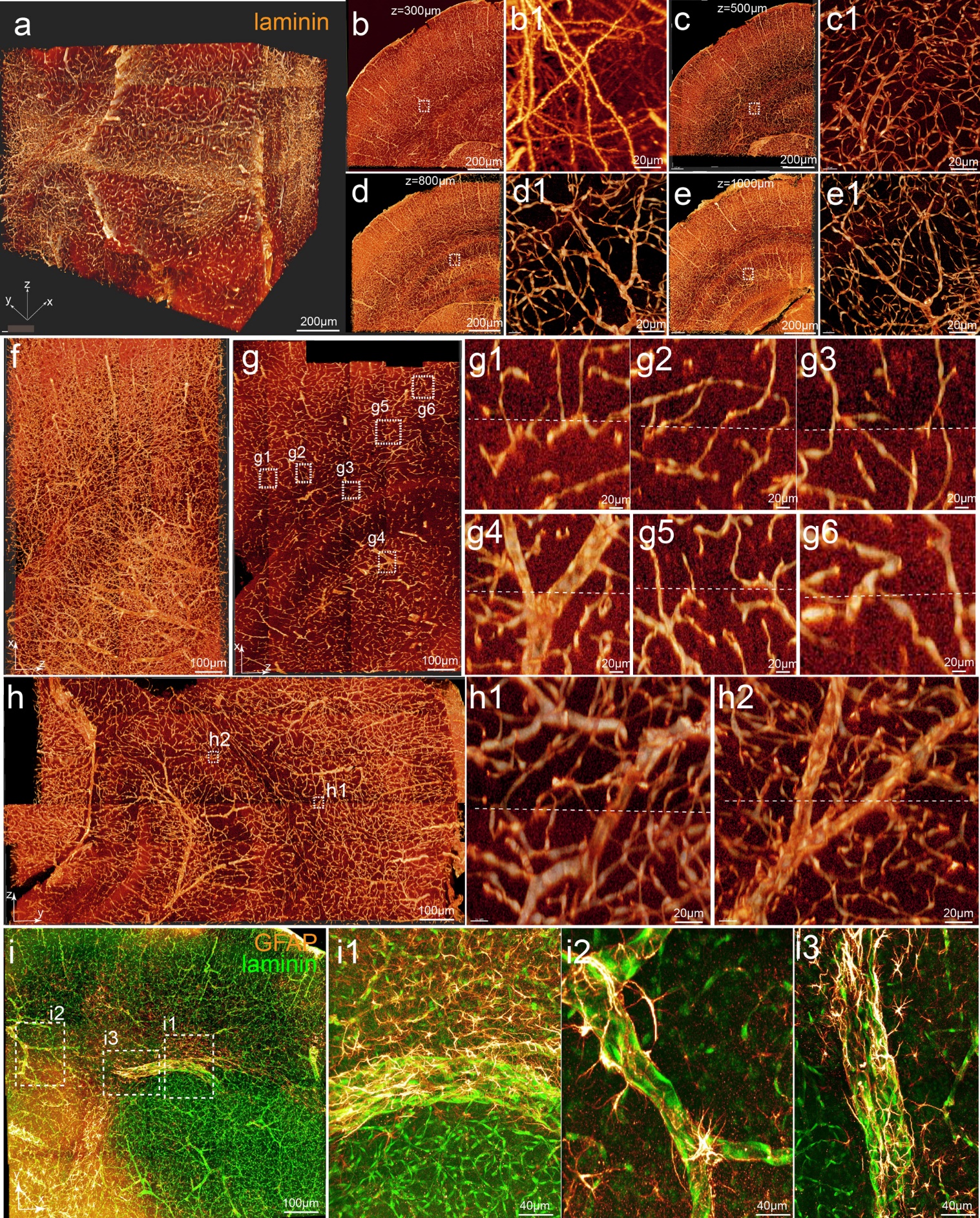


**Figure S6. High resolution imaging of brain samples after immunofluorescence labeling.** A mouse brain slice (1.5 × 1.5 × 1.0 mm^3^) was stained with antibodies against laminin (a-h) or laminin + GFAP (i), processed with the TESOS method and imaged under a confocal microscope with a 40×/1.3 NA objective (voxel size, 0.4 × 0.4 × 1.5 µm^3^).

(a). A reconstructed final image stack of 1.5 mm (x) × 1.5 mm (y) × 1.0 mm (z) was stitched from six slabs with a thickness of 200–240 µm each.

(b-e). Optical slices in the x–y orientation were acquired at various depths. Boxed regions are enlarged in (b1-e1).

(f). A sub-block of 1.5 mm (x) × 0.5 mm (y) × 1.0 mm (z) was displayed in the x–z orientation.

(g). An optical slice in the x–z orientation. Boxed regions were selected at the boundary between adjacent z-stacks and are enlarged in (g1-g6). Dotted lines indicate boundaries between two stitched adjacent stacks in the z dimension.

(h). An optical slice in the y–z orientation. Boxed regions are enlarged in (h1, h2). Dotted lines indicate boundaries between two stitched adjacent stacks in the z dimension.

(i) Reconstructed final image stack of a brain slice stained with GFAP + laminin antibodies. Boxed regions are enlarged in (i1-i3).
