# Supplementary material for: Mapping of individual sensory nerve axons from digits to spinal cord with the transparent embedding solvent system
Source: Cell Res. 2024 Jan 3;34(2):124–39. doi: 10.1038/s41422-023-00867-3 (PMC10837210; doi:10.1038/s41422-023-00867-3)
Supplement: Supplementary file 14 — Supplementary information, Figure S7 [file 41422_2023_867_MOESM14_ESM.docx]

**
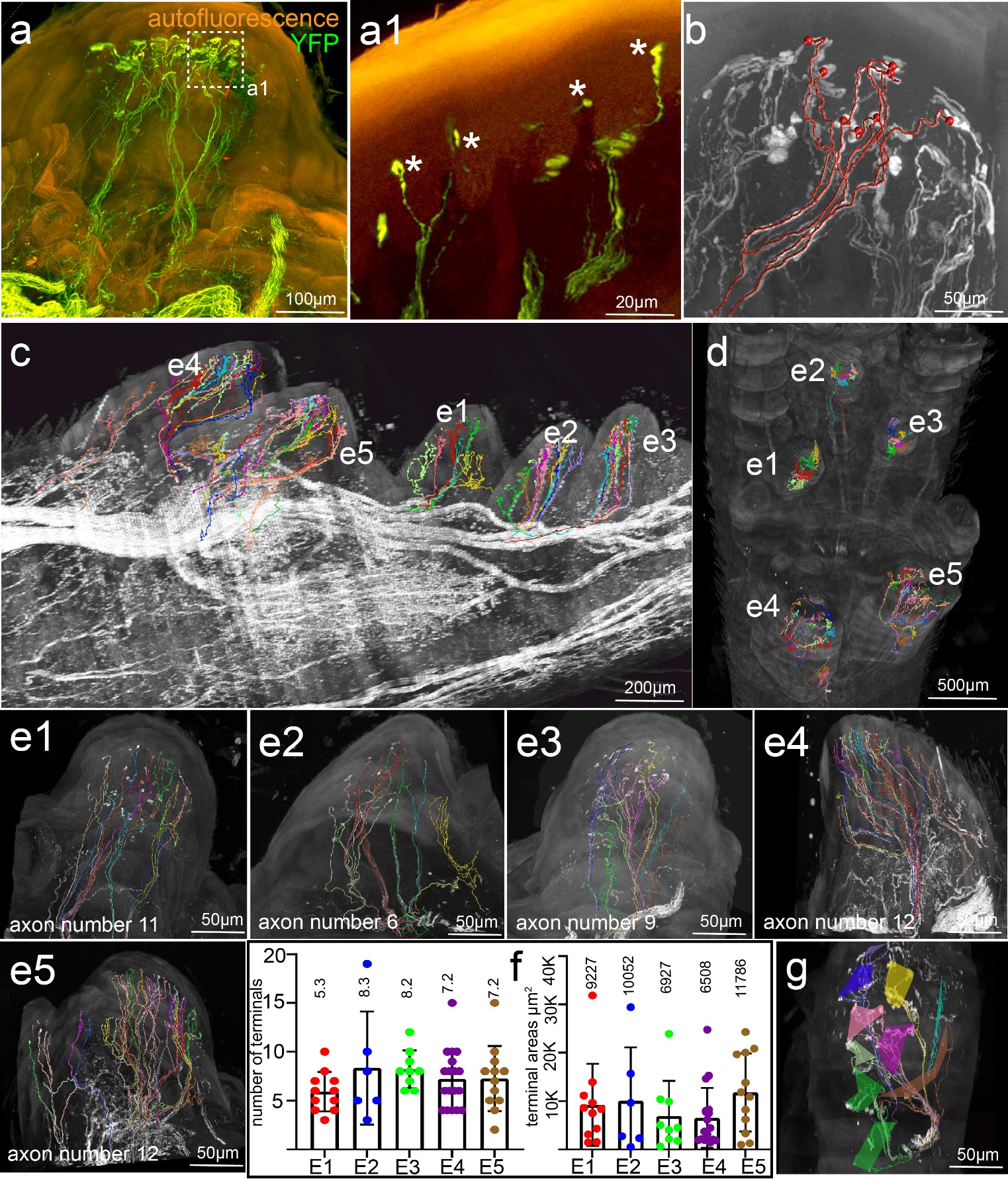
**

**Figure S7. Tracing of sensory axons innervating Meissner Corpuscles under the five walking pads of an adult *Thy1-YFP-16* mouse forepaw**.

(a). A sub-block displaying a walking pad and its innervating nerve axons. Boxed region was resliced in (a1) to show the morphology of the Meissner corpuscles (asterisks) and their localization within the dermal papillae.

(b). Tracing of one sensory axon and all of its derivative branches and terminals with Vaa3D.

(c, d). The tracing results for all the sensory axons within the five walking pads (e1- e5) are shown from the lateral (c) or ventral (d) view.

(f). Quantification of the average number of terminals associated with each sensory axon (left panel) and the average receptive field area associated with each sensory axon (right panel).

(g). The non-overlapping tiling pattern of the receptive fields associated with different sensory axons in the walking pad e1.
