# Supplementary material for: Mapping of individual sensory nerve axons from digits to spinal cord with the transparent embedding solvent system
Source: Cell Res. 2024 Jan 3;34(2):124–39. doi: 10.1038/s41422-023-00867-3 (PMC10837210; doi:10.1038/s41422-023-00867-3)
Supplement: Supplementary file 15 — Supplementary information, Figure S8 [file 41422_2023_867_MOESM15_ESM.docx]

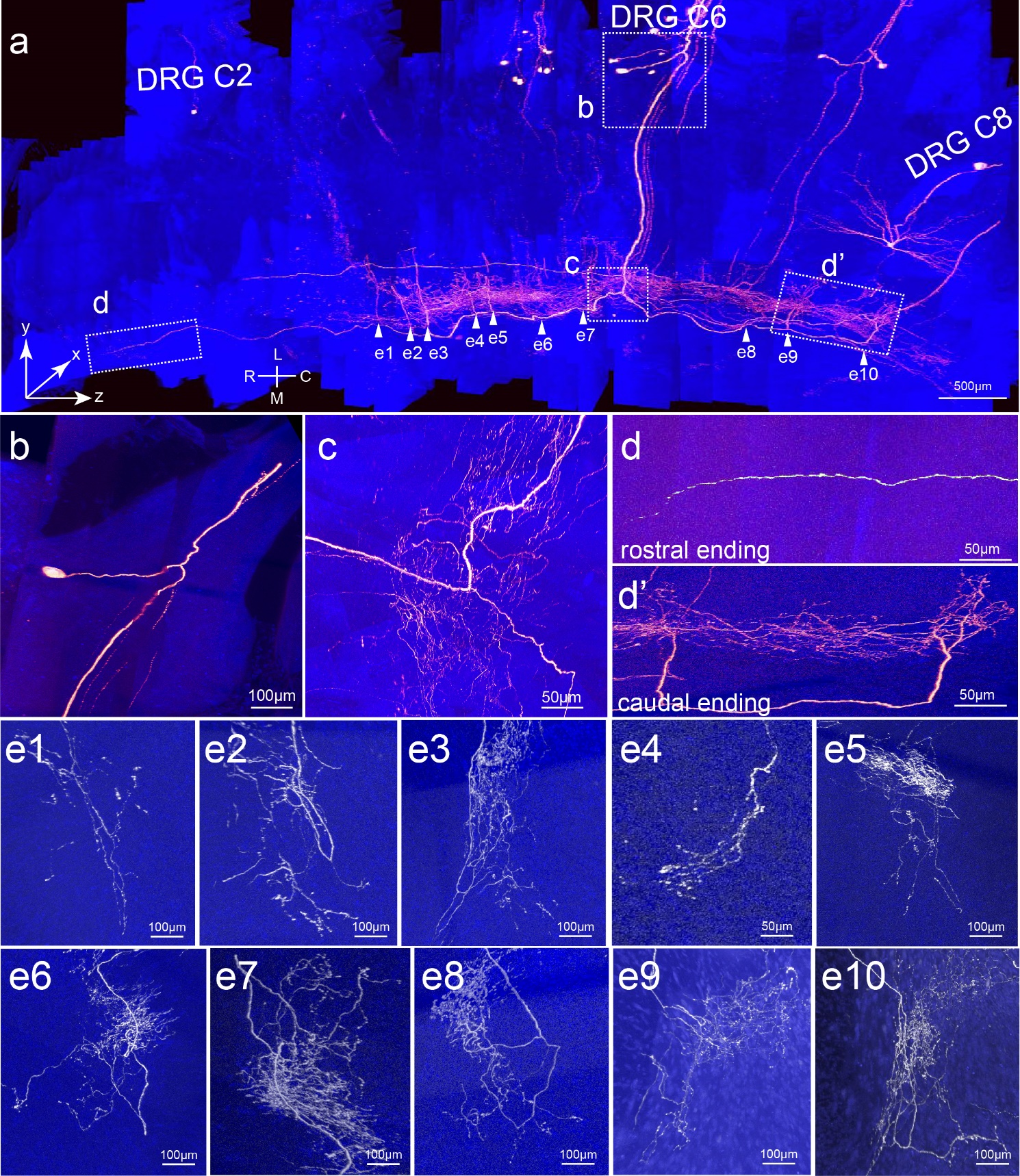


**Figure S8. Complete projection of one sensory neuron within the spinal cord.**

(a). A sensory neuron within the C6 DRG and its projection within the spinal cord are shown. Boxed regions are enlarged in the following panels.

(b). The soma and the bifurcations of the peripheral branch and central branch.

(c). The central axon branch bifurcated into caudal and rostral branches.

(d, d’). The rostral (d) and caudal (d’) termination of the axon.

(e1-e10). The 10 collateral branches of the axon and their arbors. The locations are indicated in (a).
