# Supplementary material for: Mapping of individual sensory nerve axons from digits to spinal cord with the transparent embedding solvent system
Source: Cell Res. 2024 Jan 3;34(2):124–39. doi: 10.1038/s41422-023-00867-3 (PMC10837210; doi:10.1038/s41422-023-00867-3)
Supplement: Supplementary file 16 — Supplementary information, Figure S9 [file 41422_2023_867_MOESM16_ESM.docx]

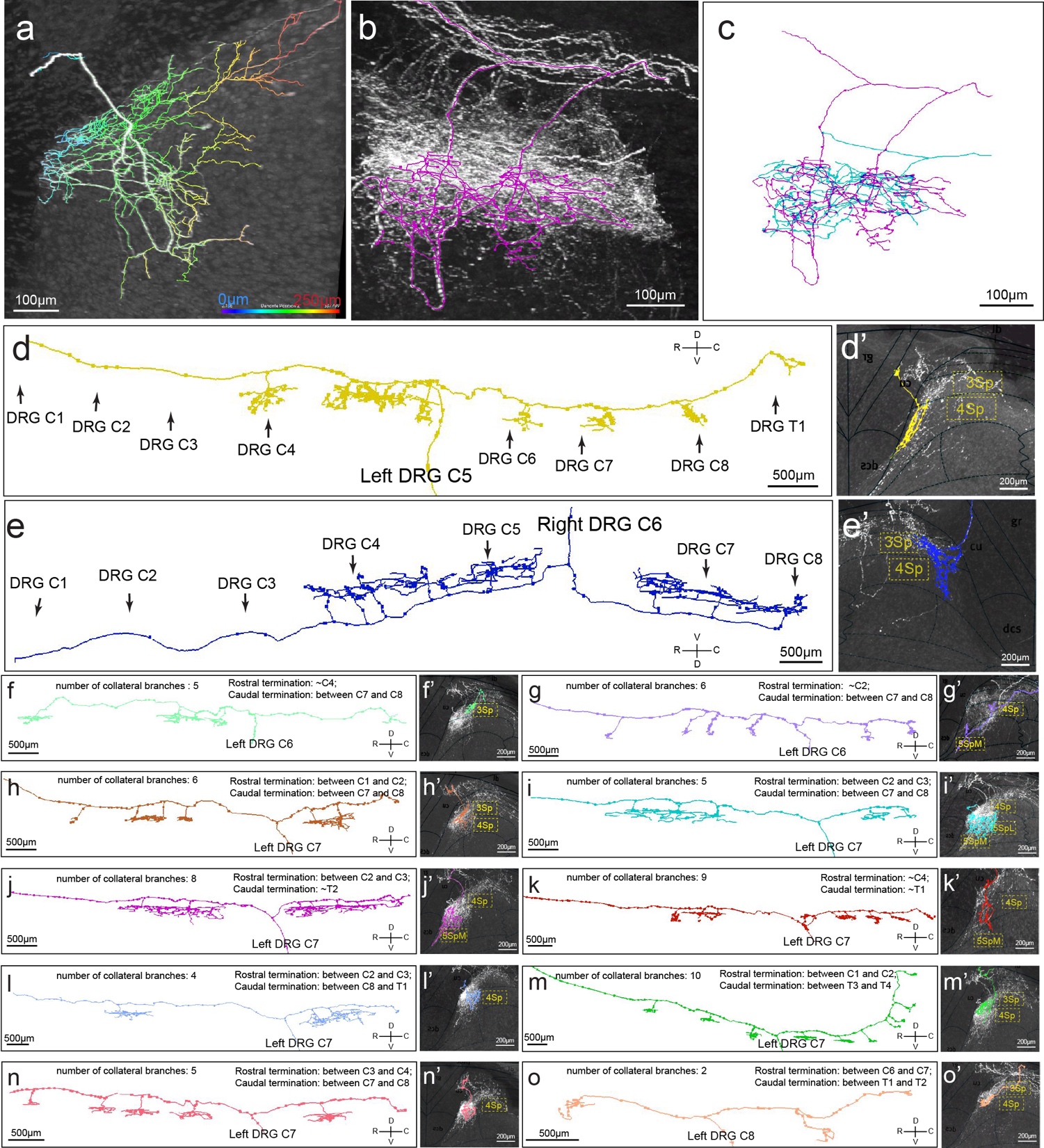


**Figure S9. Complete projection mapping of 12 sensory neurons within the spinal cord**. Axonal arbors were traced with Vaa3D.

(a). Color coded depth showing the spatial distribution pattern of an axonal arbor.

(b). Arbors from two adjacent collateral branches derived from the same neuron.

(c). Schematic of the spatial overlap of the arbors from the two neurons.

(d-o). Complete tracing of 12 sensory neurons within the spinal cord. Eleven were from the C5-C8 DRGs on the left side(d, f-o) and one was from the C6 DRG on the right side (e). (R, rostral; C, caudal; D, dorsal; V, ventral)

(d’-o’). Projections of arbors were mapped with the Allen Spinal Cord Atlas. The boxed labels indicated the projection laminae. Collateral branches number, positions of rostral and caudal terminations were described in each figure.
