# Supplementary material for: Mapping of individual sensory nerve axons from digits to spinal cord with the transparent embedding solvent system
Source: Cell Res. 2024 Jan 3;34(2):124–39. doi: 10.1038/s41422-023-00867-3 (PMC10837210; doi:10.1038/s41422-023-00867-3)
Supplement: Supplementary file 17 — Supplementary information, Data S1 [file 41422_2023_867_MOESM17_ESM.docx]

**Step-by-step Protocol of the TESOS Method**

Materials:

Quadrol: Sigma-Aldrich; 122262
Tert-Butanol: Sigma-Aldrich; 360538
Benzyl Benzoate: Sigma-Aldrich; W213802

Bisphenol-A ethoxylate diacrylate Mn 468 (BED468): Sigma-Aldrich; 413550

Irgacure 2959 (2-Hydroxy-4’-(2-hydroxyethoxy)-2-methylpropiophenone): Sigma Aldrich; 410896
EDTA-Na_2_ (Ethylenediaminetetraacetic acid disodium salt dehydrate): Sigma-Aldrich; E5134

Glass vials or 50ml conical tubes

UV curing light: Thorlabs; CS20K2

Magnetic kinematic base: Thorlabs; KB25/M or SB1/M

Solutions:

4% PFA

20% EDTA (pH=7.0-7.5)
25% w/v Quadrol solution (Quadrol is very vicious and weighing is easier)
Gradient tB delipidation solutions:

1. 30% v/v tert-Butanol + 5% w/v Quadrol
2. 50% v/v tert-Butanol + 5% w/v Quadrol
3. 70% v/v tert-Butanol + 5% w/v Quadrol

tB-Q dehydration solution: 70% v/v tert-Butanol + 30% w/v Quadrol
BB-BED clearing medium (R.I. 1.552): 47% v/v Benzyl Benzoate + 48% v/v BED468 + 5% w/v Quadrol + 2% w/v Irgacure 2959

Procedures:

1. Perfusion and tissue preparation: anesthetize mice with an intraperitoneal injection of a combination of xylazine and ketamine anesthetics (Xylazine 10-12.5 mg/kg; Ketamine, 80-100 mg/kg body weight). Inject transcardially ice-cold heparin PBS of 50-100 ml (10U/ml heparin sodium in 0.01M PBS) and then 50 ml 4% PFA.
2. Fixation: fix samples with 4% PFA at room temperature overnight. Samples can be processed in a 50 ml tube.
3. Decalcification (for hard tissue only): Immerse samples in 20% EDTA and place at 37°C on a shaker for 4 days with daily medium change. The duration of decalcification depends on the size of the bone.
4. Decolorization: Immerse samples into 25% Quadrol for 1-2 days at 37°C on a shaker for 2 days, with medium refreshed daily.
5. Delipidation and dehydration: place samples in gradient tB solutions for 1-2 days and then tB-Q for 2 days in shaker (~100rpm) at room temperature.
6. Clearing: Immerse the samples into the BB-BED clearing medium and shake it at room temperature on a shaker until transparency is reached.
7. Polymerization: place samples in disposable base molds (VWR M-475) filled with BB-BED medium. Make sure the sample is ~5mm away from the side wall. Cover the sample with a coverslip. Place the mold on ice, and irradiate it with a high-power UV curing light (Thorlabs CS20K2) with typical power of 50mW/cm^2^ for 5~10 min. Keep the lamp head 10 cm away from the sample. After polymerization, preserve the sample block in a 50ml tubes with ~3-5 ml BB-BED medium in it.
8. Sample mounting, imaging and sectioning (with a rotary microtome):
9. Use magnetic kinematic base (KB25/M or SB1/M, Thorlabs) for mounting and transferring samples.
10. Glue the embedded samples onto the top plate of the kinematic base with the epoxy resin.
11. Perform alignment to ensure the sectioning plane is parallel to the imaging plane.
12. Mount the embedded samples on the microtome and section to expose the sample top surface.
13. Image the sample with an upright confocal/2P microscopy.
14. After imaging the selected sample areas, transfer the sample to the microtome for sectioning (5-10 µm/round). The sectioning depth should be at least 10% less than the Z-stack depth to provide overlapping area for stacks stitching.
15. For tissues with strong autofluorescence, perform linear channel unmixing using the “image calculator” plugin in the Image J (NIH Image J).
16. Stitch all imaged slabs with pairwise stitching plug-in of the Image J or custom software.
17. Accomplish 3-D rendering, snapshots and animation of the stitched data with Imaris (Bitplane). Use Vaa3D for axon tracing if necessary.
