# Supplementary material for: Mapping of individual sensory nerve axons from digits to spinal cord with the transparent embedding solvent system
Source: Cell Res. 2024 Jan 3;34(2):124–39. doi: 10.1038/s41422-023-00867-3 (PMC10837210; doi:10.1038/s41422-023-00867-3)
Supplement: Supplementary file 18 — Supplementary information, Table S1 [file 41422_2023_867_MOESM18_ESM.docx]

**Table S1.** TESOS immersion method time schedule.

|  | Soft tissue organs | Hard tissue | Large body trunk |
| --- | --- | --- | --- |
| 20% EDTA | none | 4 days | 7 days |
| 25% quadrol | 2 days | 2 days | 2 days |
| 30% tert-butanol | 4 hours | 4 hours | 1 day |
| 50% tert-butanol | 6 hours | 6 hours | 1 day |
| 70% tert-butanol | 1 days | 1 days | 1 day |
| tB-Q dehydration | 2 days | 2 days | 2-3 days |
| BB-BED clearing | 1 day | 1 day | 2 days |
| Total time | 6-7 days | 10.5 days | 16-19 days |
