# Supplementary material for: Mapping of individual sensory nerve axons from digits to spinal cord with the transparent embedding solvent system
Source: Cell Res. 2024 Jan 3;34(2):124–39. doi: 10.1038/s41422-023-00867-3 (PMC10837210; doi:10.1038/s41422-023-00867-3)
Supplement: Supplementary file 19 — Supplementary information, video legend [file 41422_2023_867_MOESM19_ESM.docx]

High resolution supplementary videos (Videos 1-7) are also available online at the following Youtube channel.

<https://www.youtube.com/channel/UCtDCiJ_yAH8xAsTtK4CqTGw>

**Video 1**. Whole body imaging of a 6-week-old *Thy1-YFP-16* mouse at micrometer resolution with a light sheet microscope. Sample size 100mm × 35mm × 20mm (voxel size, 0.8 × 0.8 × 3.5 µm^3^). Red, YFP signal; green, autofluorescence. Whole body 3-D rendering data was downsampled by a factor of 3(x) × 3(y) × 2(z). Sub-blocks of craniofacial, thoracic and abdomen segments were downsampled by a factor of 3(x) × 3(y) × 1(z). Tooth sub-block was downsampled by a factor of 2(x) × 2(y) × 1(z).

**Video 2**. Whole body imaging of a mouse pup at micrometer resolution with a confocal microscope. A P5 *Thy1-YFP-16* mouse pup was processed (final sample size 35mm(x) × 10mm (y) × 18mm (z) and imaged with a 20×/0.95 NA confocal microscope (voxel size, 0.9 × 0.9 × 3.5 µm^3^). Gold, YFP signal; blue, autofluorescence). Data were downsampled by a factor of 2(x) × 2(y) × 1(z).

**Video 3**. Sub-micron resolution imaging of a *Thy1-EGFP* brain block. An adult *Thy1-EGFP* mouse brain sample of 1mm (x) X 1mm (y) X 1.5mm (z) size was processed and imaged with a 40X 1.3NA objective (voxel size 0.26µm X 0.26µm X 1.2 µm). Gold, GFP signal.

**Video 4**. Sub-micron resolution imaging of DRG sensory neurons and their projections in the spinal cord. An adult *Shh-Cre^ERT2^;Ai140* mouse cervical vertebrae segment containing the spinal cord, bones, attached muscle and skin (sample size 3.5 X 2.2 X 3mm^3^) was processed and imaged with a 40X/1.3NA confocal microscope (voxel size 0.37 × 0.37 × 1.2 µm^3^). Gold, GFP signal; blue, autofluorescence.

**Video 5**. Submicron resolution imaging of peripheral nerve axons in an intact adult *Thy1-YFP16* mouse forepaw with skin and hair. An intact forepaw (sample size 3.4 × 4.0 × 7.3mm^3^) including the skin and hair from an adult *Thy1-YFP16* mouse was imaged with a confocal microscope (voxel size 0.4 × 0.4 × 1.2 µm^3^). Yellow, YFP signal; Red, autofluorescence.

**Video 6**. Submicron resolution imaging reveals complete projections of individual sensory neurons in the spinal cord. Cervical vertebrae (2.5 × 3.8 × 6 mm^3^) with DRGs labelled with AAV was imaged with a confocal microscope (voxel size 0.4 × 0.4 × 1.2 µm^3^). Gold, GFP signal; blue, autofluorescence.

**Video 7**. Mesoscale connectome mapping of individual sensory neurons from the digit hair follicles on the forepaw to the spinal cord. Body segment from an adult *Thy1-EGFP* mouse (25 × 18 × 20mm^3^) was processed and imaged with a confocal microscope (voxel size 0.4 × 0.4 × 1.5 µm^3^).
